# γδ T cells show distinct responses to CMV after stem cell transplantation

**DOI:** 10.1007/s00109-026-02703-8

**Published:** 2026-07-20

**Authors:** Freya Sibbertsen, Zheng Song, Cedric Ly, Inga Sandrock, Stefan Bonn, Christian Koenecke, Likai Tan, Christian Schultze-Florey, Immo Prinz

**Affiliations:** 1 Institute of Systems Immunology, University Medical Center Hamburg-Eppendorf, Hamburg, Germany; 2 Hamburg Center for Translational Immunology (HCTI), University Medical Center Hamburg-Eppendorf, Hamburg, Germany; 3 Center for Molecular Neurobiology Hamburg (ZMNH), University Medical Center Hamburg-Eppendorf, Hamburg, Germany; 4 Institute of Medical Systems Bioinformatics, University Medical Center Hamburg-Eppendorf, Hamburg, Germany; 5 Institute of Immunology, Hannover Medical School, Hannover, Germany; 6 Deutsches Zentrum für Kinder- und Jugendgesundheit (DZKJ), Hamburg, Germany; 7 Center for Biomedical AI, University Medical Center Hamburg-Eppendorf, Hamburg, Germany; 8 Department of Hematology, Hemostasis, Oncology, and Cell Therapy, Hannover Medical School, Hannover, Germany; 9 Department of Anaesthesia and Intensive Care and Peter Hung Pain Research Institute, The Chinese University of Hong Kong, Hong Kong, Hong Kong SAR, China; 10 Li Ka Shing Institute of Health Sciences, The Chinese University of Hong Kong, Hong Kong, Hong Kong SAR, China

**Keywords:** γδ T cells, Cytomegalovirus reactivation, TCR sequencing, scRNA-seq, non-Vγ9Vδ2 γδ T cells

## Abstract

**Abstract:**

Cytomegalovirus (CMV) reactivation is a frequent complication after allogeneic hematopoietic stem cell transplantation (aHSCT) and critically shapes immune reconstitution. However, the clonal and functional dynamics of T cell responses to CMV remain insufficiently defined. In this study, we combined longitudinal single-cell RNA sequencing with paired T cell receptor sequencing to track γδ and αβ T cell clones at clonal resolution across five post-transplant time points in patients with and without CMV reactivation. This integrative approach revealed marked, patient-specific expansion of non-Vγ9Vδ2 γδ T cell clones, particularly within Vδ1⁺ and Vδ3⁺ subsets, which was associated with differentiation toward cytotoxic and antiviral effector states characterized by IFN-γ and TNF-α expression. Conventional CD8⁺ αβ T cells showed comparatively modest clonal dynamics during CMV reactivation in this cohort. Longitudinal tracking of individual clonotypes demonstrated heterogeneous but recurrent trajectories, with expanding γδ T cell clones frequently acquiring antiviral and cytotoxic phenotypes following CMV reactivation. These findings highlight the adaptive-like behavior and functional plasticity of non-Vγ9Vδ2 γδ T cells and provide a high-resolution framework for studying antiviral immune responses during immune reconstitution.

**Key messages:**

- Single-cell sequencing enables clonal tracking of T cells during CMV reactivation.
- γδ T cells show patient-specific clonal expansion after CMV reactivation.
- Expanding γδ T cell clones acquire antiviral and cytotoxic phenotypes.
- Vγ9Vδ2 γδ T cells remain stable with limited clonal expansion.
- αβ T cells display limited clonal responses during CMV reactivation.

**Graphical Abstract:**

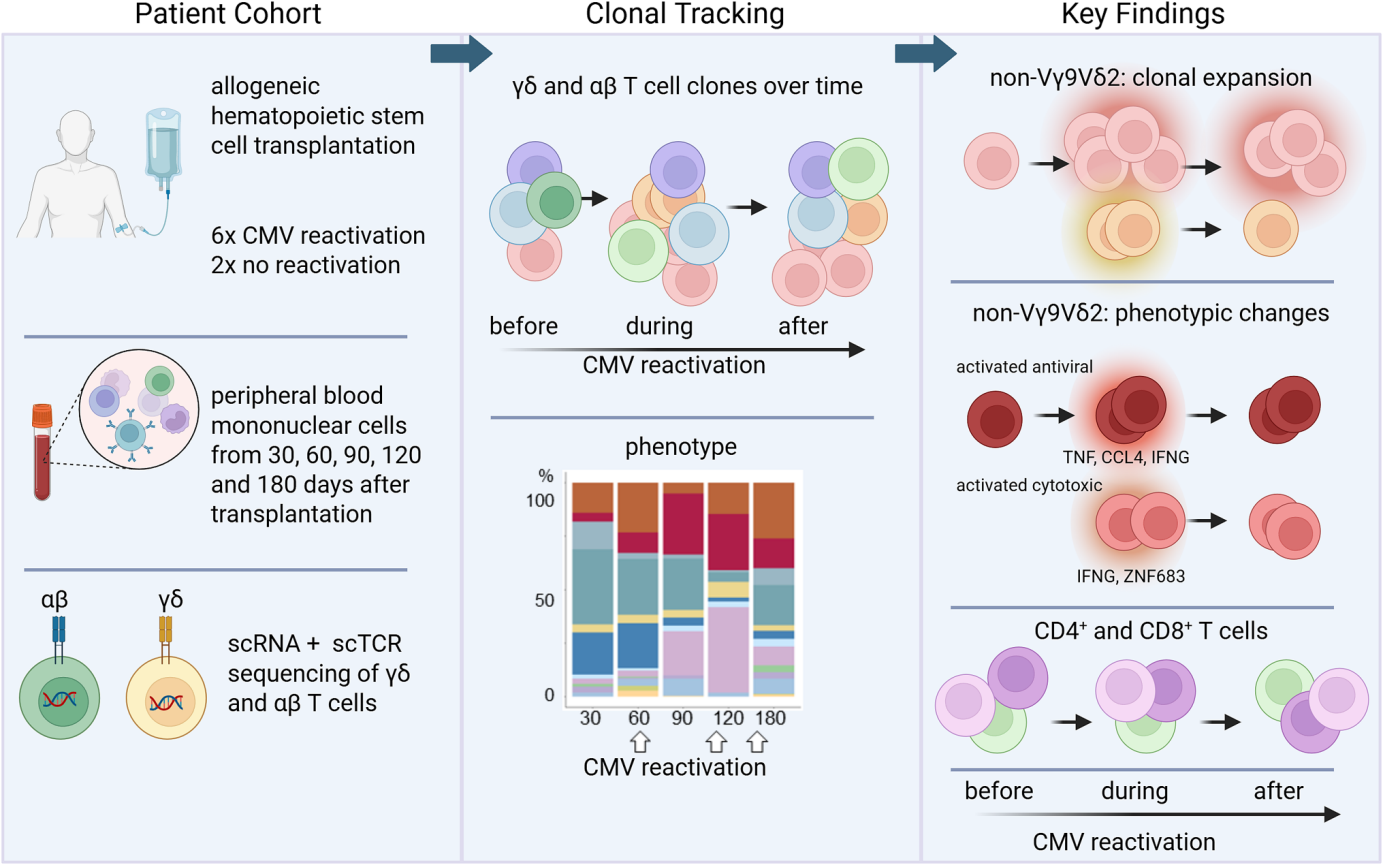

**Supplementary Information:**

The online version contains supplementary material available at 10.1007/s00109-026-02703-8.

## Introduction

While cytomegalovirus (CMV) infection and latency pose limited risk to immunocompetent individuals, immunosuppressed individuals are at high risk for a more severe course of infection or viral reactivation, particularly in the context of allogeneic hematopoietic stem cell transplantation (aHSCT). Consequently, in aHSCT, CMV serostatus of donor (D) and recipient (R) determines the risk and nature of viral reactivation. In D +/R + settings, latent CMV in both donor- and recipient-derived cells can reactivate under post-transplant immunosuppression, thereby influencing immune reconstitution and T cell dynamics [1]. At the same time, antiviral responses of the newly reconstituted immune system are an essential component contributing to effective management of CMV infection[2]. This highlights the need to understand the immune responses involved in CMV reactivation.

γδ T cells, which are regarded as “unconventional” T cells as opposed to “conventional” αβ T cells, reconstitute faster after aHSCT[3], and their early presence after immunodepletion has been associated with favorable outcomes in transplantation settings[4–10]. γδ T cells act mostly independent of peptide/MHC presentation and upon activation, they can be cytotoxic and release inflammatory cytokines[11–13]. The most abundant γδ T cell subset in the human blood are innate-like Vγ9Vδ2^+^ T cells, which uniformly expresses a semi-invariant T cell receptor (TCR) composed of a Vγ9 and a Vδ2 TCR chain. The canonical Vγ9Vδ2^+^ TCR is interacting with phosphoantigen-activated butyrophilin molecules on the surface of target cells[14–18]. In contrast, other γδ T cells expressing different γδ TCR chain combinations such as, e.g., Vδ1^+^ or Vδ3^+^ TCRs are collectively designated as non-Vγ9Vδ2 T cells. There is evidence that non-Vγ9Vδ2 T cells are directly involved in the immune defense against CMV in transplant patients[19, 20]. Bulk TCR sequencing revealed that these cells can expand in a clonal fashion in response to CMV infection, which is therefore regarded as an adaptive-like immune response[21–23]. Thus, CMV-induced effector γδ T cells may directly control viral infection by eliminating infected cells and by inhibiting viral replication[24–26].

However, it is unclear how distinct types of γδ T cells respond to CMV reactivation on a clonal level. To address this, we used single cell RNA and TCR sequencing (scRNA-seq and scTCR-seq) to track individual γδ and αβ T cell clones in the peripheral blood of patients undergoing aHSCT before, during, and after CMV reactivation. TCR sequences served as barcodes to longitudinally track T cell phenotype and proliferation at clonal resolution. We show that CMV-induced expansion of non-Vγ9Vδ2 T cells was accompanied by acquisition of anti-viral effector functions in aHSCT patients, whereas innate-like Vγ9Vδ2^+^ T cells and also CD8^+^ αβ T cell clones remained less responsive.

## Results

### Tracking γδ T cell dynamics in response to CMV reactivation

**Fig. 1:**
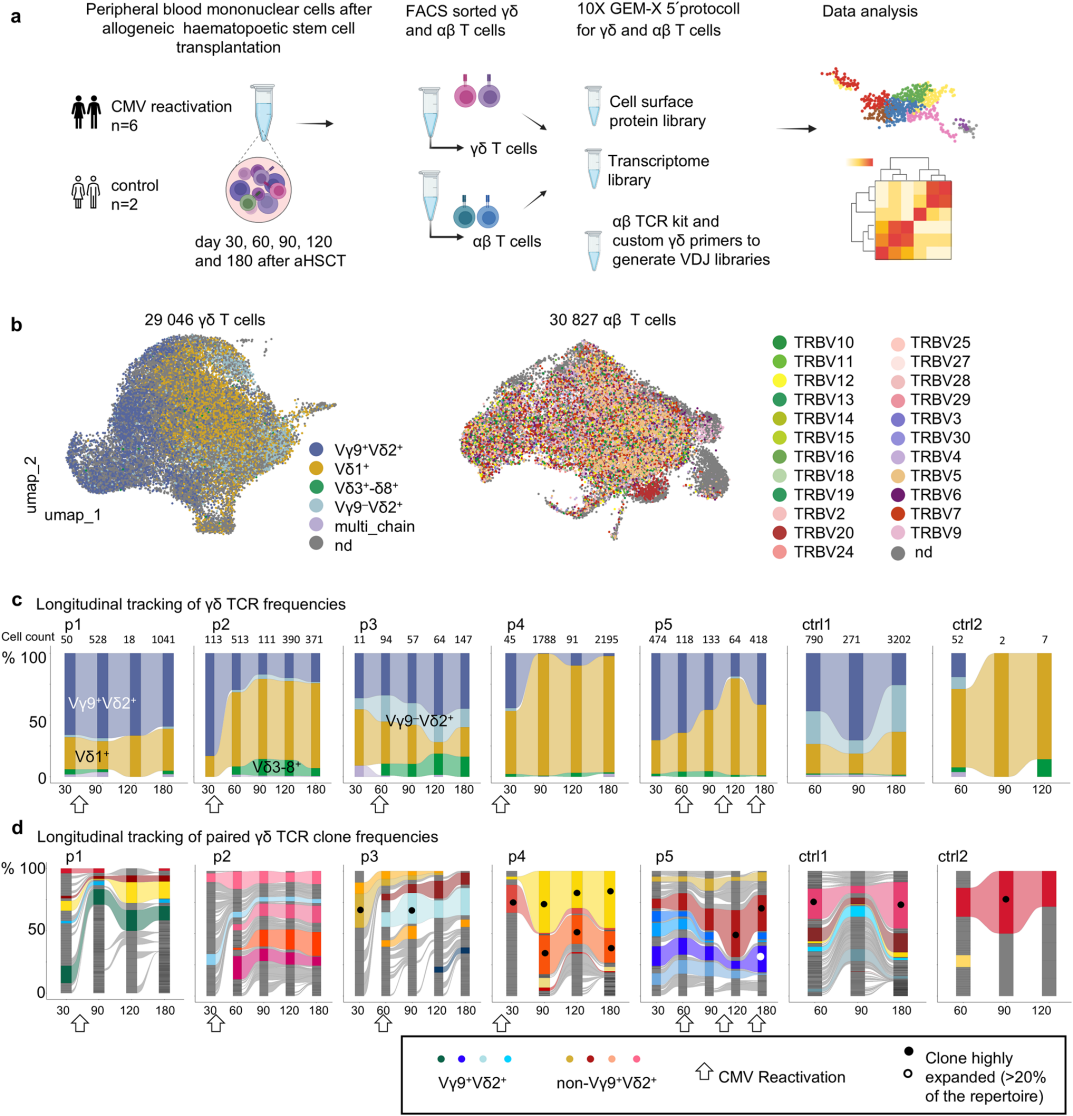
**Longitudinal tracking and distribution of γδ T cells in response to CMV reactivation reveals a shift toward Vδ1**^**+**^** expanding γδ T cells. a**) Schematic overview of the workflow: αβ and γδ T cells were sorted from human peripheral blood mononuclear cells (PBMCs) using fluorescence-activated cell sorting (FACS). Sorted cells were then subjected to a modified version of the 10 × genomics 5′ protocol to amplify the CDR3δ and CDR3γ regions using primers that were previously described for the TRGC and TRDC segments. The flow chart was created with BioRender.com. **b**) Two-dimensional visualisation (UMAP) of sorted γδ and αβ T cells recovered after sort and quality control. Integrated data from all patients was clustered and projected onto a two-dimensional approximation. Cells are coloured based on their usage of the delta or beta chain. **c**) Frequency of paired Vγ9Vδ2^+^, Vδ1^+^, Vδ3–8^+^ and non-Vγ9Vδ2 γδ clones per patient longitudinally. **d**) Longitudinal analysis of paired γδ T cell receptor (TCR) usage across different patients at various time points following transplantation. Each panel represents the relative frequency of specific TCR clones for patients with and without CMV reactivation. The black and white arrow indicates CMV reactivation and black circles indicate clones of interest that are highly expanded (> 20% of the repertoire at the indicated timepoint)

Blood samples were obtained in a prospective cohort study to assess T cell responses to CMV in patients undergoing aHSCT at Hannover Medical School from 2014 to 2016. Importantly, Letermovir CMV prophylaxis was not yet standard of care at that time. CMV reactivation was tightly monitored, and patients were preemptively treated with Ganciclovir and/or Valganciclovir. Peripheral blood samples were obtained on day 30, 60, 90, 120, and 180 post aHSCT. To compare the αβ and γδ T cell responses to CMV at the clonal level, we sorted αβ and γδ T cells from aHSCT patients with (n = 6) or without (n = 2) episodes of CMV reactivation and performed single cell sequencing for RNA expression (scRNAseq), cell surface protein (CSP), as well as γδ and αβ TCR repertoires (scTCRseq). For each patient, sorted T cells from all-time points were hash-tagged and pooled to preclude batch effects. The workflow is shown in Fig. 1a.

After quality control, a total of 29,385 γδ T cells and 30,827 αβ T cells from eight donors for five timepoints were identified by scRNA-seq profiles as described[27] (Supplementary Fig. 2a, Supplementary Table 3). We successfully mapped 20,791 *TRD* chains and 16,078 *TRG* chains to the transcriptome data, enabling us to identify 13,163 cells with γδ TCR pairs. No clones with identical paired *TRG* and *TRD* sequences were shared between individual patients. However, in line with previous reports[23, 28], we observed “public” *TRGV9* sequences that were overlapping between patients, resulting in more inter-individual overlap of the *TRG* than of the *TRD* repertoires (Supplementary Fig. 2b). Due to the limited number of recovered γδ cells, p6 was excluded from further analysis for γδ T cells (Supplementary Table 3). For αβ T cells, we mapped 14,408 *TRA* chains and 17,579 *TRB* chains to the transcriptome data and identified 13,925 paired αβ TCRs. Overall, both the α and β TCR chain sequences were hardly overlapping between patients, comparable to the rather “private” δ TCR chain repertoires (Supplementary Fig. 2c, d).

By mapping the VDJ information onto the transcriptome data, we could further annotate Vγ9Vδ2^+^, other Vδ2^+^ (Vγ9^–^Vδ2^+^), as well as Vδ1^+^ and Vδ3^+^ γδ T cells and visualize their location within the two-dimensional UMAP (Fig. 1b, left panel). This showed a distinct distribution of γδ T cell subsets expressing either *TRDV1* or *TRDV2*. At the same time, αβ T cells expressing various *TRBV* chains were homogeneously distributed across the corresponding UMAP (Fig. 1b, right panel). Following CMV reactivation, we observed a heterogenous increase in Vδ1^+^ and other non-Vγ9Vδ2 γδ T cells, which was accompanied by a decrease of Vδ2^+^ γδ T cells by for most but not all patients (Fig. 1c). Flow cytometry validation suggested that average frequencies of non-Vδ2 γδ T cells significantly increased over time after aHSCT (Fig. 1c, right panel, Supplementary Fig. 3). Next, we quantified the frequency of each γδ T cell clone per timepoint per patient among all paired clones. In patients p1—p5, multiple new γδ T cell clones emerged and expanded after CMV reactivation. Clones constituting more than 20% of the repertoire were categorized as highly expanded (Fig. 1d). Similar results were obtained when analyzing only the individual CDR3 regions of δ clones (Supplementary Fig. 4a). The emergence of new non-Vγ9Vδ2 clones was also evident by smaller (< 0.5) Morisita-Horn indices indicating less clonal overlap in the repertoire between the timepoint before and after CMV reactivation, while later time points shared most clones (p2,3,4 Supplementary Fig. 4b). TCR repertoire diversity, measured by Gini index, confirmed inter-patient heterogeneity across timepoints, while overall clonal dominance progressively increased from early to late post-transplant timepoints. Patient4 demonstrated a massive clonal expansion, suggesting a strong antigen-driven response, likely reflecting CMV-driven T cell proliferation following viral reactivation. In contrast, most patients maintained relatively stable moderate clonality, although a general trend toward higher Gini indices was observed across all patients over time (Supplementary Fig. 4c). The T cell repertoire in control patients remained stable in γδ T cells with most clones being not or lowly expanded and only a few clones dominating the repertoire without changes in the frequency (Fig. 1d and Supplementary Fig. 4). Together, these results underscore the adaptive-like immune response of non-Vγ9Vδ2 T cells during CMV reactivation. However, individual dynamics of γδ T cells were heterogenous in each patient.

### Differentiation of γδ T cell clones towards antiviral and cytotoxic phenotypes after CMV reactivation

**Fig. 2:**
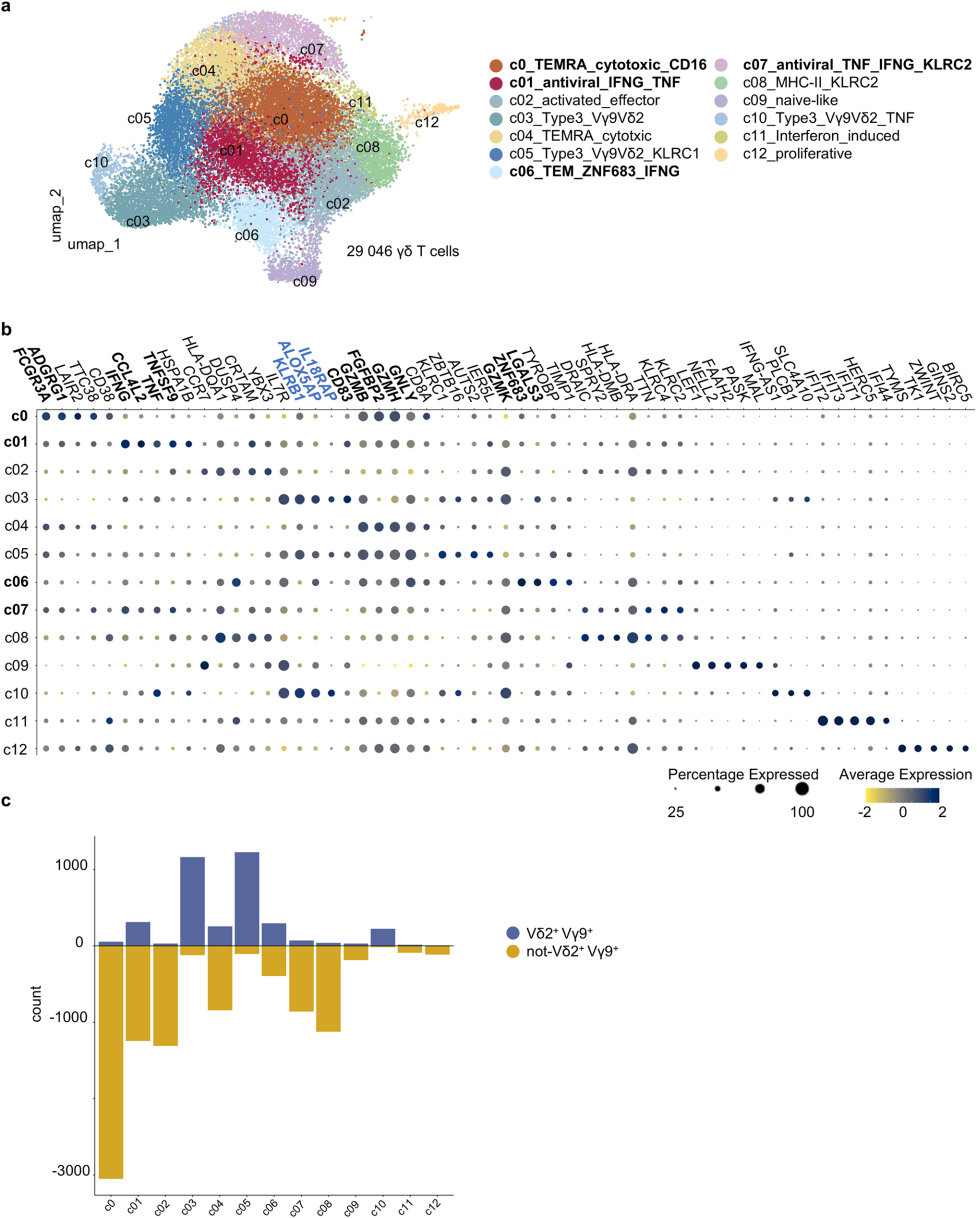
**Phenotype of γδ T cells longitudinally. a**) Two-dimensional visualisation (UMAP) of sorted γδ T cells recovered after sort and quality control. Integrated data from all patients was clustered and projected onto a two-dimensional approximation. Clustertree was used to determine the correct resolution to identify clusters. Cells are coloured based on the cluster. **b**) Phenotype of γδ T cell clusters. Differentially expressed genes (DEG) per cluster defining the phenotype of each cluster after exclusion of T cell receptor genes. In bold relevant genes of cluster of interests. **c**) Distribution of clusters longitudinally of all five patients with CMV reactivation combined. Bar charts showing the phenotype of all-γδ, non-Vγ9Vδ2 and Vγ9Vδ2^+^ of each patient individually can be found in Supplementary Fig. 5, 6 and 7, respectively

The integrated data of seven recipients were clustered based on the expression of highly variable genes, projected in two dimensions using UMAP, displaying the distribution of cells for each patient separately (Fig. 2a, Supplementary Fig. 5a- d). We identified 13 clusters labeled c0 to c12 (Fig. 2b) that were annotated based on differential expressions of differentiation, activation and linage markers and/or transcription factors and previous annotations [29, 30]. Non-Vγ9Vδ2 T cells dominated clusters such as cluster c0 presenting a cytotoxic *ADGRG1, FCGR3A, FGFBP2, GZMH* and *GZMB* Temra phenotype. Expression of FGFBP2 further indicated differentiation toward a terminal effector memory (TEMRA) phenotype [31]. Antiviral clusters were characterized by expressions of *CCL4L2*, *CCL4*, *IFNG*, and *TNF*, consistent with a highly activated effector profile [29, 32, 33] (clusters c01 and c07). Vγ9Vδ2^+^ T cells were most commonly present in cluster c03, c05, and c10. These clusters are defined by markers such as *IL7R*, *KLRB1*, *IL18RAP* and *CD83* and present a Type-3 immunity (Type3_Vγ9Vδ2 [30]) phenotype. Interestingly, clusters 4 and 6 turned out to be heterogeneous clusters with both Vγ9Vδ2^+^ and non-Vγ9Vδ2 T cells. Both clusters are defined by cytotoxic modulators such as *GZMB*, *GZMH* and *GNLY*, with cells in cluster c06 expressing further *ZNF683*, *GZMK* as well as *IFNG* (Fig. 2c, Supplementary Fig. 5e).

Non-Vγ9Vδ2 T cells predominantly presented a cytotoxic phenotype prior and following CMV reactivation (c0), and shortly after aHSCT, they could be further identified in antiviral clusters c01, and c07. This trend was observed in all patients with CMV reactivation, however, in p2 and p3 to a lesser extent. In p2 and p3, most cells were identified in clusters c04, c06 and c08, showing higher cytokine expression but less CTL activity (Supplementary Fig. 6).

As expected, Vγ9Vδ2^+^ T cells show little variation in response to cytomegalovirus (CMV) reactivation (Supplementary Fig. 7). However, despite the antigen-presenting capability suggested by *CD83* [34] expression and expression of cytotoxic mediators, Vγ9Vδ2^+^ T cells did not undergo significant clonal expansion (Fig. 1e), even after antigen exposure[35]. (Supplementary Fig. 7).

In summary, the analysis of the phenotype in the CD3^+^ T cell repertoire in this patient cohort revealed clonal expansions and phenotypic shifts in non-Vγ9Vδ2 T cells following CMV reactivation. Meanwhile, Vγ9Vδ2^+^ T cells dominated early post-aHSCT, maintaining a Type 3 phenotype. While many γδ clones showed a similar frequency and phenotype during the course of reactivation, non-Vγ9Vδ2 T cells displayed more dynamic changes, transitioning to antiviral and cytotoxic profiles in response to CMV reactivation. These data underscore the individualized immune response to CMV and highlight the adaptive-like capabilities of γδ T cells in response to CMV reactivation.

### Vδ1 γδ T cell clones show a cytotoxic phenotype after CMV reactivation

We aimed to evaluate the phenotype and dynamics of paired and single γδ TCR chains over time. As no paired clonotypes were shared between patients, we used the paired γ/δ TCR sequence as a unique barcode to track individual clones longitudinally. This allowed us to distinguish expanding from non-expanding clones and to assess whether expansion was accompanied by phenotypic changes.

**Fig. 3:**
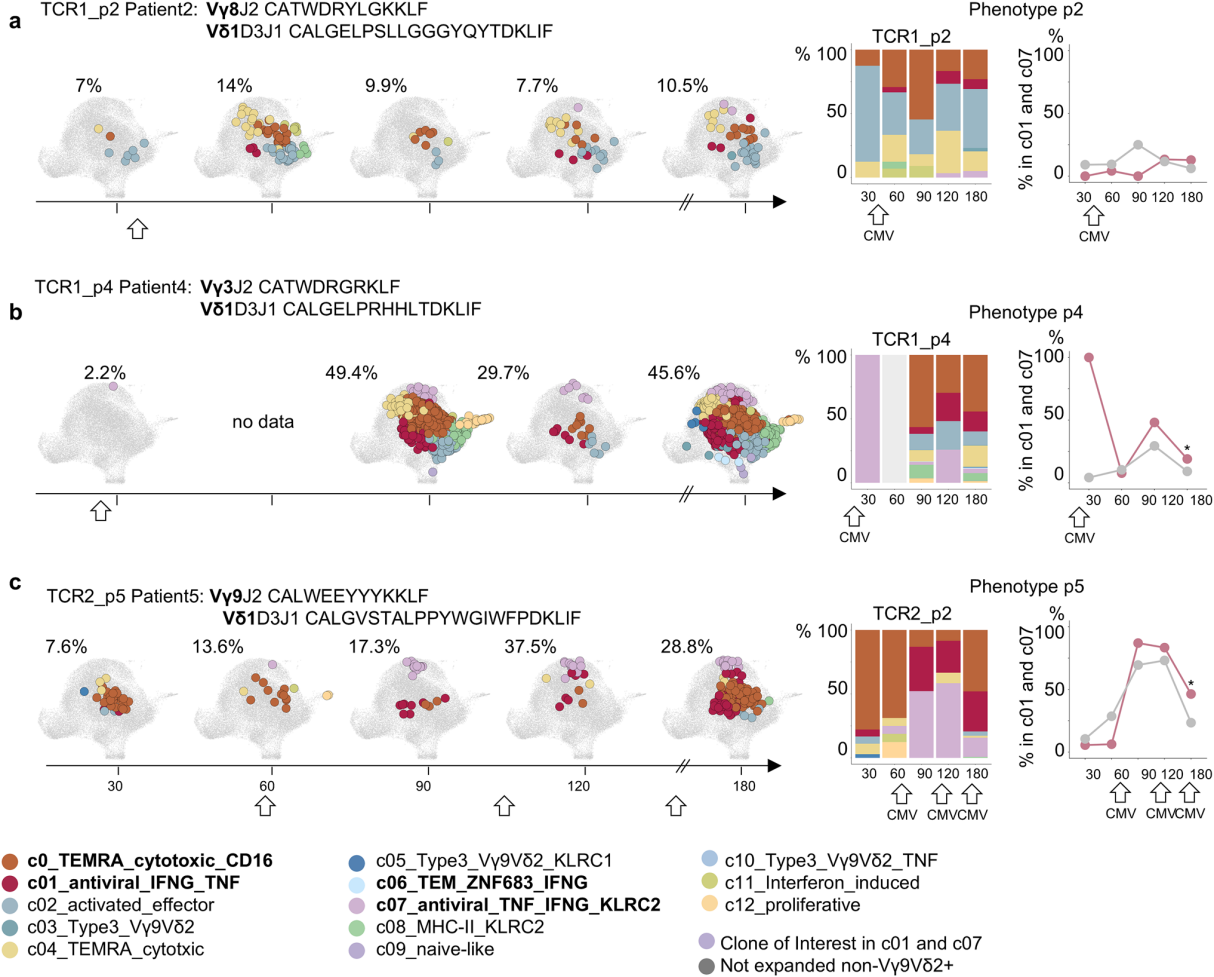
**Clonal expansion and phenotypic shift of three representative clones towards a TNF**^**+**^** and IFN-γ**^**+**^** antiviral phenotype of non-Vγ9Vδ2 T cells.** Phenotype of three individual clones of interest from **a**) patient2, **b**) patient4 and **c**) patient5. Tracking the phenotype of the clone of interest longitudinally in the UMAP. Highlighted dots represent one cell of the indicated clone. All clones share the same paired CDR3 sequence. The frequency of the clone of interest per patient per day is indicated above each UMAP. Bar chart representation of the phenotype of the clone of interest longitudinally (left side). Line plots show the proportion of cells within the cluster (c01 and c07) for clones of interest (red) versus all other clones (gray) across post-transplant timepoints. Significance stars indicate Bonferroni-adjusted Fisher's exact test p-values (*p < 0.05) (right side). Cells are coloured base on the cluster they´re identified (Fig. 2a). The black and white arrow indicates CMV reactivation. Grey bars indicate no sample available

Across several patients, CMV reactivation was associated with clonal expansion and clear transcriptional shifts. We defined expanding clonotypes as those showing at least a two-fold increase in relative abundance compared to the preceding time point after CMV reactivation. These clones often transiently dominated the repertoire and displayed marked changes in phenotype (Fig. 3).

We first identified an antiviral, TCR-driven γδ T cell phenotype characterized by expression of *CCL4*, *TNF* and *IFNG* (clusters c01 and c07; Fig. 3a-c). The most abundant clone in p2 (TCR1_p2, Vγ8Vδ1^+^ TCR Fig. 3a) doubled the frequency after CMV reactivation and showed an increase presence in clusters c0 and c01 with reduced representation in c02. The most prominent expansion and phenotypic shift was observed in p4 with one Vγ3Vδ1^+^ clone rapidly expanding after CMV reactivation dominating the repertoire and another Vγ3Vδ1^+^ clone dominating the repertoire that was not identified at day 30 post-aHSCT (TCR1_p4, Fig. 3b and TCR2_p4 Supplementary Fig. 8a). After CMV reactivation both clones were predominantly identified in cluster c0 and antiviral cluster c01 and c07 and present in the proliferating cluster c12. (Fig. 3b). With multiple CMV reactivations of p5 and changes in the phenotype towards an antiviral phenotype in all γδ cells, we expected similar changes to see on a single-clone level. The second-most abundant clone (TCR2_p5, Vγ9Vδ1, Fig. 3c) was particularly noteworthy; prior to infection, it was localized in the CD16^+^ TEMRA cytotoxic (cluster c0) cluster and remained there during the first reactivation without exhibiting clonal expansion (Fig. 3c). However, following the first and second reactivation, this clone was mainly identified within the *TNF*, *IFNG* and cytotoxic cluster (c01 and c07). After the third reactivation, it was found in the antiviral and cytotoxic clusters (c0, c01 and c07). A Vγ3 and Vγg9 Vδ1 showed a similar pattern of increased presence in antiviral clusters and after the first CMV reactivation contraction of the antiviral phenotype **(**TCR3_p5, Supplementary Fig. 8b).

When comparing phenotypic patterns, we observed that the expanded clones of interest are showed concordant phenotypic patterns with the non-expanded non-Vγ9Vδ2 clones in each patient during or following CMV reactivation but maintain the increased antiviral phenotype longer than non-expanded clones.

**Fig. 4:**
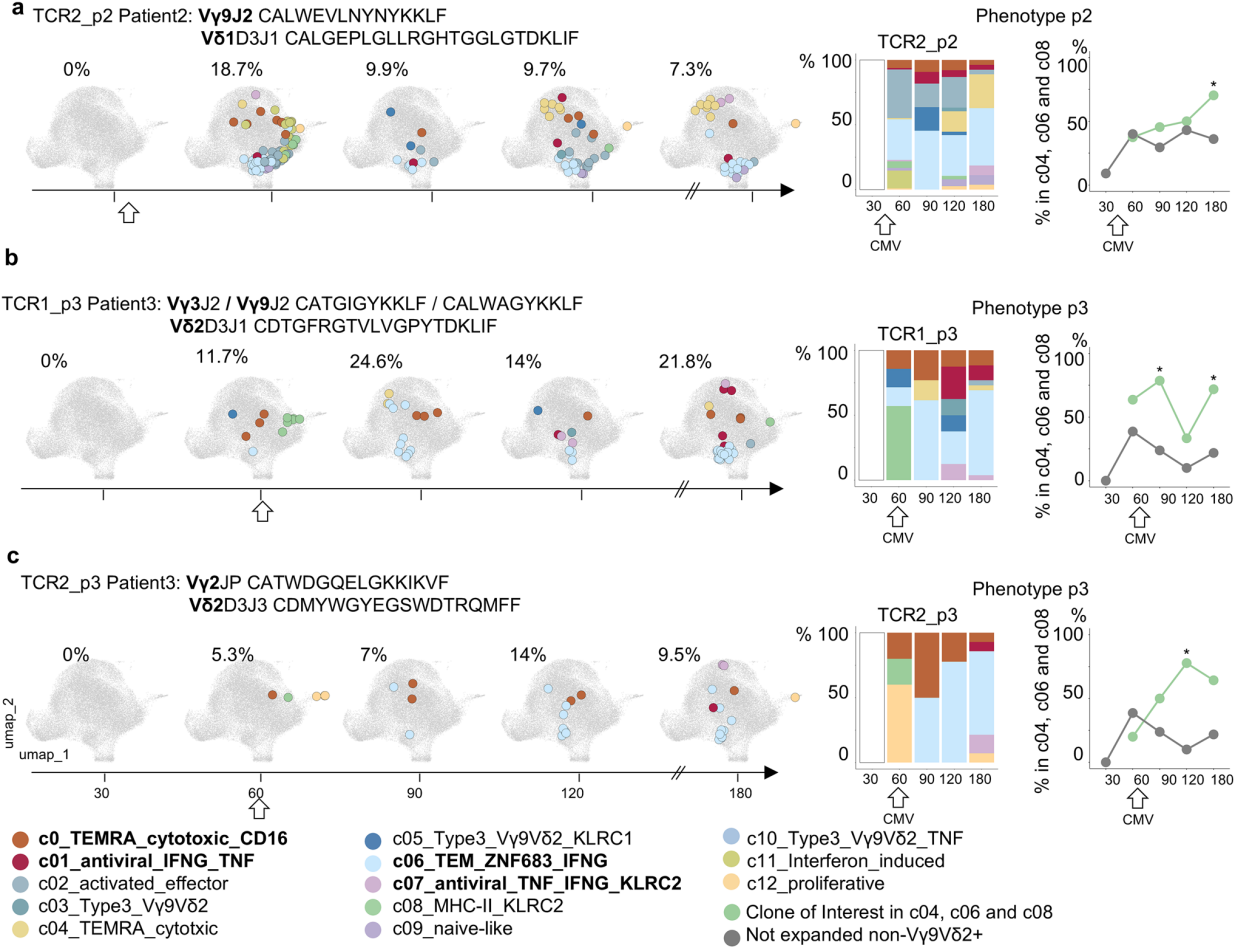
**Clonal expansion and phenotypic shift of three representative clones towards an IFN-γ**^**+**^** and ZNF683**^**+**^** phenotype.** Phenotype of three individual clones of interest from **a**) patient2 and **b-c**) patient 3. Tracking the phenotype of the clone of interest longitudinally in the UMAP. Highlighted dots represent one cell of the indicated clone. All clones share the same paired CDR3 sequence. The frequency of the clone of interest per patient per day is indicated above each UMAP. Bar chart representation of the phenotype of the clone of interest longitudinally (left side). Line plots show the proportion of cells within the cluster (c04, c06 and c08) for clones of interest (green) versus all other clones (grey) across post-transplant timepoints. Significance stars indicate Bonferroni-adjusted Fisher's exact test p-values (*p < 0.05,) (right side). Cells are coloured base on the cluster they´re identified (Fig. 2a). The black and white arrow indicates CMV reactivation. Grey bars indicate no sample available

Next, we identified expanded γδ T cell clones in response to CMV reactivation that were predominantly localized in memory T cell clusters c04, c06 and c08, expressing *GZMH, GZMK, IFNG, GNLY, STAT1/5, HLA-Ds, LGALS3* and *ZNF683.* While one clone from patient 2 (TCR1_p2) presented a *TNF*-driven phenotype, multiple clones emerged after CMV reactivation including TCR2_p2 (Vγ9Vδ1) and TCR3_p2 (Vγ2Vδ1). These clones were identified shortly after CMV reactivation and are later predominantly present in clusters c04 and/or c06 (Fig. 4a, Supplementary Fig. 8c). Surprisingly, we observed clonal expansion of a Vγ9Vδ2^+^ clone after CMV clearance that had an additional Vγ3 chain (TCR1_p3, Fig. 4b). Notably, the *TRGV9* rearrangement used a *J2* segment rather than a *TRJP* TCR gene segment. A similar pattern of phenotypic shift and expansion were observed for the TCR2_p3 (Vγ2Vδ2, Fig. 4c) clones. These clones appeared in MHC-II and KLRC2^+^ (c08), cytotoxic TEMRA (c04), and TEM_ZNF683_IFNG (c06) clusters during reactivation and were predominantly found in c06 thereafter. Following clearance, they were also detected in c0 and c01.

When comparing the phenotype of each expanded clone to the phenotype of the non-expanded non-Vγ9Vδ2 clones, clusters c04, c08 and especially, c06, defined by *ZNF683* and *IFNG*, seems overrepresented in these clones that are dominating the repertoire of each patient. Following CMV reactivation, these clones of interest were significantly more frequent in clusters c04, c06, and c08 compared to all non-expanded clones from the same patient. These results provide robust statistical support for the phenotypic shift toward antiviral and cytotoxic states in expanded non-Vγ9Vδ2 clones after CMV reactivation.

**Fig. 5:**
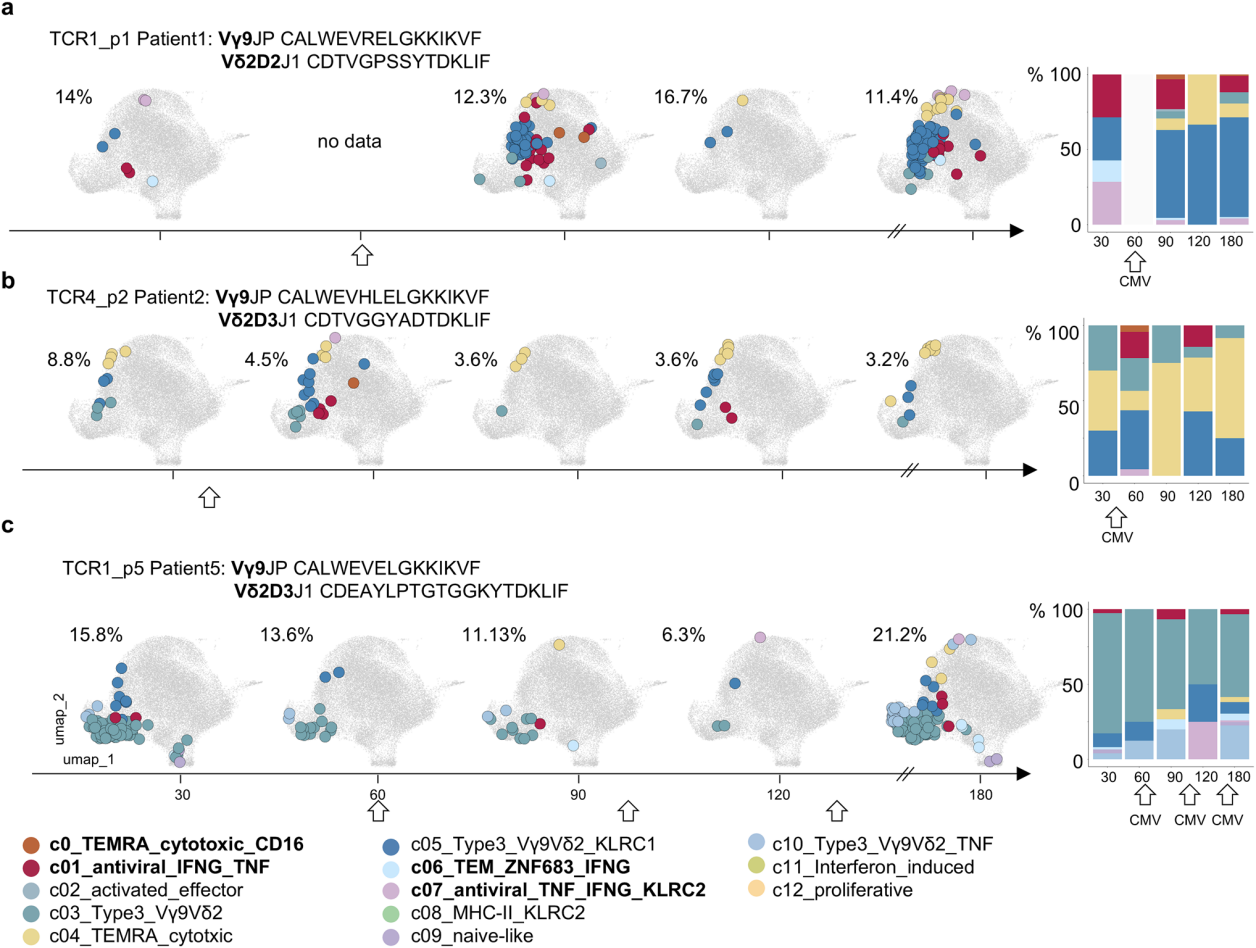
**Phenotype of three representative clones of Vγ9Vδ2**^+^ **γδ T cells.** Phenotype of three individual clones of interest from patient1(**a**), patient 2(**b**) and patient5(**c**). Tracking the phenotype of the clone of interest longitudinally in the UMAP. Highlighted dots represent one cell of the indicated clone. All clones share the same paired CDR3 sequence. The frequency of the clone of interest per patient per day is indicated above each UMAP. Bar chart representation of the phenotype of the clone of interest longitudinally. Cells are coloured base on the cluster they´re identified (Fig. 2a). The black and white arrow indicates CMV reactivation. Grey bars indicate no sample available

In contrast, we did not expect to see phenotypical changes in genuine innate-like Vγ9JPVδ2^+^ T cells (Fig. 5). Accordingly, TCR1_p1, TCR4_p2 and TCR1_p5 (all Vγ9JPVδ2, Fig. 5a-c) were neither expanding during CMV reactivation nor changing their phenotype. TCR1_p1 (Vγ9JPVδ2) contracted after CMV reactivation and expanded after clearance. This clone was present in the clusters c03, c04 and c05 before and after CMV reactivation (Fig. 5). Similarly, in control patients without CMV reactivation, dominant γδ clones (ctrl1 and ctrl2) showed neither expansion nor phenotypic shift (Supplementary Fig. 8d-e).

Together, these findings show that CMV reactivation induced dynamic, clone-specific changes within the γδ T cell compartment. Expanding clones frequently acquire antiviral and cytotoxic phenotypes, whereas innate-like Vγ9JPVδ2^+^ clones remain largely stable. This highlights the functional plasticity and heterogeneity of γδ T cell clones in adapting to viral challenges.

### Conventional CD4^+^ and CD8^+^ T cells are comparably less active during CMV reactivation in bone marrow transplant patients

**Fig. 6:**
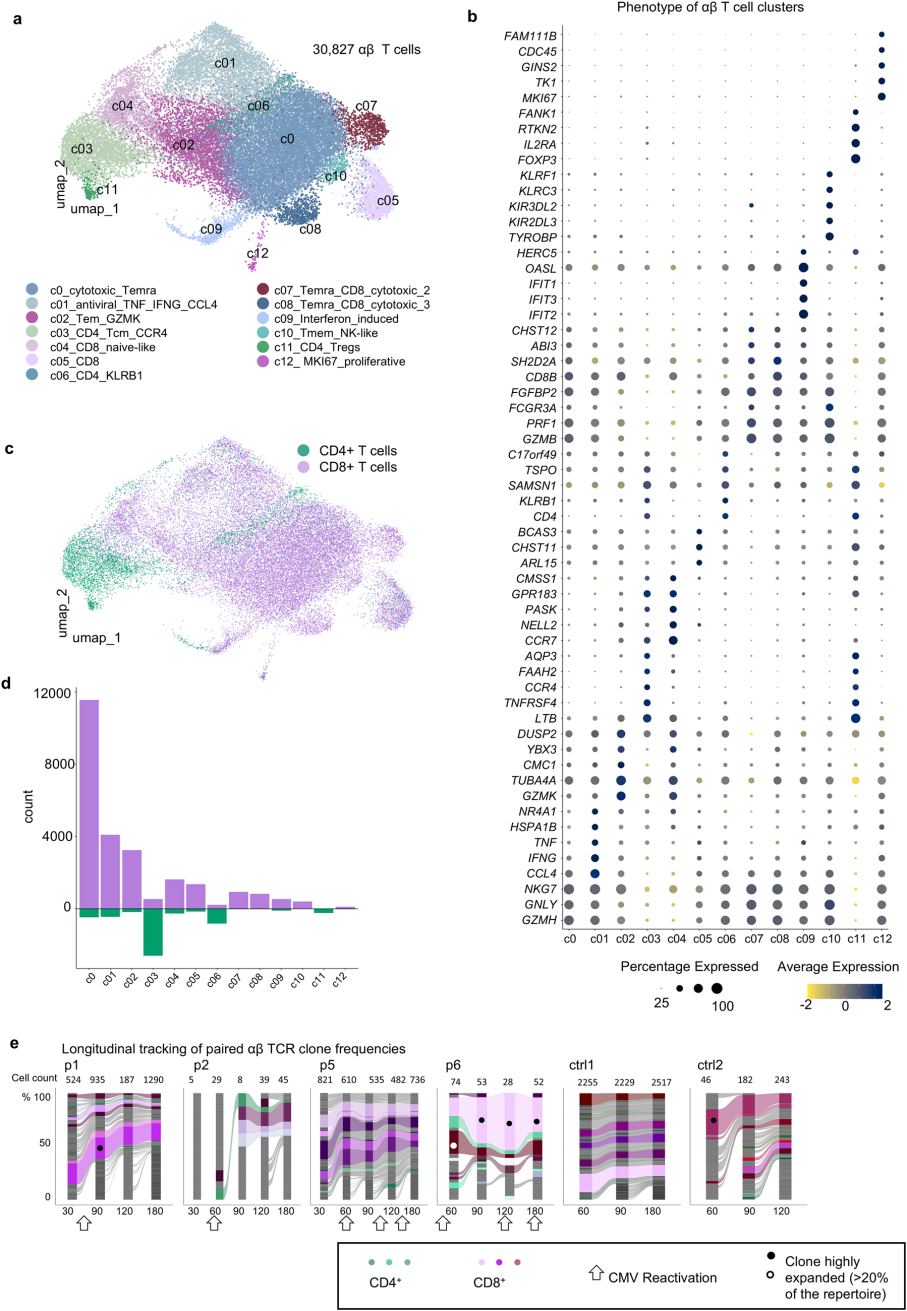
**Phenotype of αβ T cells longitudinally. a**) Visualization of the two-dimensional reduction after sort and quality control of 30827 αβ T cells recovered from six patients (control = 2, CMV reactivation n = 4). Integrated data from all patients was clustered and projected onto a two-dimensional approximation. Clustertree was used to determine the correct resolution to identify clusters. Cells are coloured based on the cluster. **b**) Differentially expressed genes (DEG) per cluster defining the phenotype of each cluster after exclusion of T cell receptor genes. In bold relevant genes of cluster of interests. **c**) Distribution of CD4^+^ und CD8^+^ T cells on the UMAP. CD4^+^ und CD8.^+^ T cells were annotated based on gene expression and surface expression of CD4 and CD8. **d**) Longitudinal analysis of paired αβ T cell receptor (TCR) usage across different patients at various time points following transplantation. Each panel represents the relative frequency of specific TCR clones for patients with and without CMV reactivation. The orange triangle indicates CMV reactivation and black circles indicate clones of interest that are highly expanded (> 20% of the repertoire at the indicated timepoint)

We simultaneously analyzed CD4^+^ and CD8^+^ T cells in the context of CMV reactivation in the same patients (p1, p2, p5, p6, ctrl1, ctrl2). We identified 13 clusters in αβ T cells that were annotated based on gene expression and surface proteins (Fig. 6a, b). CD4^+^ T cells were located in the left half of the UMAP and were either annotated as central memory T cells (Tcm) or CD4^+^ Treg. Cytotoxic CD4^+^ T cells were located between or in clusters dominated by cytotoxic CD8^+^ T cells (Fig. 6c). CD4^+^ and CD8^+^ clusters were predominantly separated (Fig. 6d). CD4^+^ T cells, especially CD4^+^ Tregs, were displaying a low clonality, possibly a result of their later reconstitution after aHSCT (Fig. 6e).

Following CMV reactivation, multiple new paired αβ T cell clones were identified, analogous to the γδ T cell response described above. In two patients with a single reactivation before day 60, multiple new clones emerged post-reactivation. CMV reactivation in p6 occurred prior to the first availability of cells. Interestingly, in p5, where multiple Vδ1^+^ clones were expanding after CMV reactivation, no αβ T cell clones were prominently expanding. The only highly expanded clone was one CD8^+^ T cell clone identified in p6, which dominated the repertoire during recurrent reactivation. Other patient samples showed no signs of clonal expansion among their CD8^+^ αβ T cells, rather dominant clones defined the repertoire without obvious changes in response to or during CMV reactivation (Fig. 6e).

Regarding CD4^+^ T cells, we only identified one clone among highly expanded clones in p6, which could also be a result of the low number of cells recovered from this patient (Supplementary Table 3).

In our longitudinal cohort, individual patient responses to CMV reactivation varied (Supplementary Fig. 9, 10). Patient1 showed stable αβ T cell phenotypes without significant clonal changes. In contrast, p2 exhibited a substantial decrease in CD4^+^ Tcm cluster alongside an increase in cytotoxic and antiviral αβ T cell populations in CD8^+^ T cell-dominated clusters until day 90, after which the phenotype stabilized. p5 showed an increase in cells within the antiviral cluster following the first CMV reactivation, indicating a reactive but transient adaptive response. Similarly, p6 showed an elevation in αβ T cells within the antiviral cluster after a second reactivation, suggesting a specific yet delayed clonal response. As expected, no phenotypic shift was observed in both control patients.

**Fig. 7:**
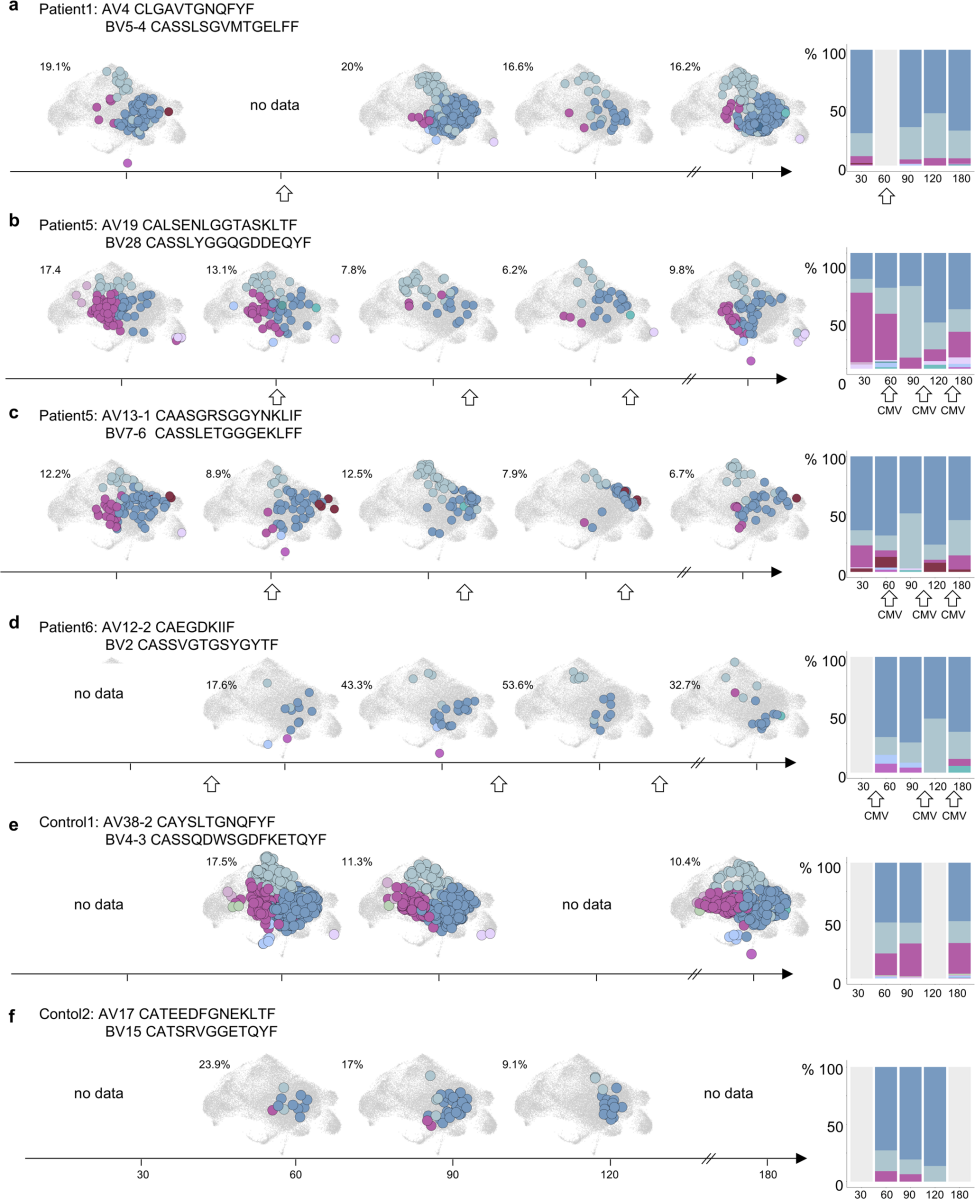
**Phenotype of six representative clones of CD8**^**+**^** T cells.** Phenotype of six individual clones of interest from **a**) patient1, **b-c**) patient5, **c**) patient6, **d**) control1 and **e**) control2. Tracking the phenotype of the clone of interest longitudinally in the UMAP. Highlighted dots represent one cell of the indicated clone. All clones share the same paired CDR3 sequence. The frequency of the clone of interest per patient per day is indicated above each UMAP. Bar chart representation of the phenotype of the clone of interest longitudinally. Cells are colored base on the UMAP cluster they are mapped to (Figure 6a). The black and white arrow indicates CMV reactivation. Grey bars indicate no sample available

In sum, striking expansions of αβ T cell clone frequencies were not observed following CMV reactivation (Fig. 7, Supplementary Fig. 10d). E.g., in p5 we did not observe clonal expansion despite experiencing multiple reactivations.

## Discussion

In this study, we demonstrate clonal expansion and phenotypic adaptation of individual γδ T cells in response to CMV reactivation post aHSCT. Our findings are consistent with previous studies reporting clonal expansion of Vδ1⁺ γδ T cells after CMV reactivation using bulk γδ TCR sequencing approaches[23]. The integration of scRNA-seq and scTCR-seq can provide valuable insights into T cell responses in diverse contexts, including tumor immunity after immune checkpoint inhibition, autoreactive T cell responses in inflammatory diseases, and antiviral immune responses[36–38]. Here, we apply this approach to identify clonal behavior and phenotypic trajectories of γδ T cells during CMV reactivation in aHSCT patients. Across the six patients experiencing CMV reactivation, we observed a relatively heterogeneous γδ TCR repertoire. This variability highlights pronounced interpatient heterogeneity in γδ T cell responses to CMV reactivation.

γδ, especially non-Vγ9Vδ2 γδ T cells, showed a robust and evident immune response to CMV[23, 26, 39–43]. It is evident that TCR-dependent recognition of γδ T cells is an essential part of the γδ T cell immune response[25]. Clonal expansion of single clones in response to CMV infection has been already described[23, 44]; however, the heterogenous phenotypes of expanding clones have not been described yet. Highly expanded γδ T cell clones accounted for more than 20% of the clonal repertoire at specific time points in multiple patients. These clones showed adaptive-like T cell response to CMV reactivation. During and after CMV reactivation, these expanded clones, particularly in patients p4 and p5, were predominantly found in cytotoxic and antiviral clusters (c01 and c07). Across all time points, most γδ T cells were assigned to cluster c0, which displayed a cytotoxic phenotype marked by expression of CD8, CD16, granzymes, granulysin, and perforin[29]. These observations are consistent with previous reports describing expanded, activated, and highly cytotoxic Vδ1⁺ γδ T cells in CMV-viremic patients.

In contrast to non-Vγ9Vδ2 γδ T cells, Vγ9Vδ2⁺ cells did not display collective clonal expansion or long-lasting phenotypic shifts following CMV reactivation. Our data are consistent with these findings and suggest that Vγ9Vδ2⁺ γδ T cells contribute to the CMV response in a limited and indirect manner[35].

Interestingly, we identified a single Vδ2⁺ clone in patient p3 that expanded after CMV reactivation and acquired a TEM_ZNF683_IFNG (c06) phenotype. This clone expressed both a Vγ3 and a Vγ9 chain and was not assigned to Vγ9Vδ2^+^-dominated clusters. Instead, it clustered with Vδ1⁺ cells in the c06 memory cluster and the c0 TEMRA cytotoxic cluster and therefore exhibited rather adaptive-like behavior and functional properties. Notably, the Vγ9 chain of this clone was not joined to JγP but instead utilized a Jγ2 segment. Functionally, non–JγP-connected Vγ9Vδ2⁺ γδ T cells share greater similarities with adaptive-like non-Vγ9Vδ2 γδ T cells than with canonical Vγ9Vδ2⁺ cells[45–47].

The target antigens recognized by expanding non-Vγ9Vδ2 γδ T cells during CMV reactivation remain incompletely understood. Previous studies have identified MHC and MHC-like molecules as γδ TCR ligands in the context of CMV infection. For example, the target of a highly expanded clone in patient p4 has been identified as HLA-DR[13], while endothelial protein C receptor (*EPCR*) has been described as a ligand for an expanded Vγ4Vδ5⁺ clone during primary CMV infection after lung transplantation[44, 48].

In addition to TCR-mediated recognition, NK related markers may contribute to γδ T cell activation and expansion. Recognition of CMV peptide–HLA-E complexes via NKG2C (*KLRC2*)[49] represents one possible mechanism. In our dataset, we observed *KLRC2* expression after CMV reactivation, particularly in clusters c06, c07, and c08, most prominently in patient p5, who experienced multiple CMV reactivation episodes. This pattern suggests that NKG2C expression may be induced following initial activation by other stimuli, including γδ TCR engagement.

Although γδ T cell repertoires gradually reconstitute after aHSCT[23], the composition of the graft strongly influences early immune responses to infections[50]. A higher frequency of γδ T cells in the graft has been associated with strong anti-leukemia effects, while γδ T cells have also been implicated in alloreactivity and the development of acute graft-versus-host disease[51, 52]. CD8⁺ γδ T cells derived from CMV-seropositive grafts preferentially adopt a TEMRA phenotype, show enhanced activation, and respond more rapidly to TCR stimulation [53]. Conversely, Vγ9Vδ2⁺ γδ T cells have been associated with a lower incidence of GvHD and viral infections in pediatric aHSCT, underscoring functional differences between γδ T cell subsets[54].

Adaptive-like γδ T cells share several features with CD8⁺ αβ T cells, including clonal expansion, memory differentiation, and effector phenotypes[22, 55]. However, in our cohort, we did not observe a strong sustained αβ T cell response to CMV reactivation. This may reflect the faster kinetics of αβ T cell responses, which could have been missed by our sampling intervals[56], or, more likely, immunosuppressive therapy may impose a stronger impact on αβ T cell activation and antiviral functions than on γδ T cells[57]. Thus, while Kaminski et al. demonstrated a dose-dependent but incomplete inhibitory effect of immunosuppression on non-Vδ2 γδ T cells [41], γδ T cells could still serve as a functional bridge, providing early antiviral immunity between weaning of immunosuppression and recovery and reconstitution of conventional αβ T-cell responses. In conclusion, γδ T cells are heterogenous and this study provides further evidence for the dichotomic role of γδ T cells. While the majority of Vγ9Vδ2⁺ cells displayed no traceable responses to CMV reactivation, we observed dramatic phenotypic changes after CMV reactivation of non-Vγ9Vδ2 T cells towards an antiviral phenotype. The observed interpatient heterogeneity of non-Vγ9Vδ2 γδ T cell clones reacting to CMV reactivation is further proof for the adaptive-like features of this subset. While some previous studies suggested significant clonal expansion following CMV reactivation and primary infection, we observed this phenomenon in only a subset of patients. It is important to consider that aHSCT-induced lymphopenia may result in a limited clonal diversity and pool of T cells capable of responding to CMV reactivation. This limitation may have influenced the degree of clonal expansion observed in our study.

## Limitations of the study

First, our study focusses solely on patients with D +/R + serologies, which may limit the generalizability of the results. CMV-naïve γδ and αβ T cells may have different responses than those observed in previously infected populations, potentially influencing the overall dynamics of the immune response. In addition, not all patients and time points were available for analysis, which may affect the comprehensiveness of our findings. Second, performing comprehensive multi-omics scRNA-seq and scTCR-seq for an in-depth analysis of γδ and αβ T cell responses to CMV reactivation over five timepoints after aHSCT came at the cost of a limited cohort size of eight patients including only one control with sufficient counts of γδ and αβ T cell for all subsets with VDJ information. Therefore, our study is not powered to correlate clonal T cell responses with clinical outcomes. The results nevertheless provide an overview of how individual γδ and αβ T cell clones respond to CMV challenges in aHSCT patients in an unpreceded resolution.

Finally, this study focused exclusively on γδ and αβ T cells, without addressing the role of the innate immune system. In particular, other immune cell populations such as natural killer cells, which share the expression of multiple functional and immunologically relevant markers with γδ T cells, were not evaluated.

## Methods

### Study design and ethics

Human blood samples were obtained from aHSCT patients between 2014 and 2016 at the Department of Hematology, Hemostasis, Oncology, and Stem Cell Transplantation, Hannover Medical School (MHH) The study was performed (in accordance with the declaration of Helsinki) and approved by the institutional review board at Hannover Medical School (#1303–2012, #2032–2013, #2604–2014, and #2604–2015), and written informed consent was obtained from all participants. EDTA blood samples were collected during routine checkups at Hannover Medical School (Table 1). PBMCs were isolated with Ficoll-Paque density-gradient media (GE Healthcare) and resuspended in PBS with 1% FCS for immediate use or frozen in RPMI with 10% FCS, 100 U/ml penicillin, 100 µg/ml streptomycin sulfate, and 10% DMSO for long-term storage.

**Table 1:** Patient characteristics

Patients (n = 8)	
Female (in %)	4 (50%)
Recipient age at aHSCT	57.5 (± 6) years
Donor age at donation	42.1 (± 12.4) years
R +/D +	8 (100%)
Graft: peripheral blood stem cells	8 (100%)
Acute GvHD	4 (50%)
Chronic GvHD	2 (25%) after sampling phase
First CMV reactivation	34.8 ± 11.7 days post-Tx
Myeloablative conditioning	3 (37.5%)
Transplant Indication	
Acute myeloid leukemia	5 (62.5%)
Myelodysplastic syndromes	1 (12.5%)
Diffuse large B-cell lymphoma	1 (12.5%)
Acute lymphoblastic leukemia	1 (12.5%)
T-cell depletion	
ATG	6 (75%)
Thymoglobulin, post-Tx Cyclophosphamide	each 1 (12.5%)

### Study cohort and experimental strategy

This study included eight patients undergoing aHSCT at the MHH clinic Department for Hematology, Hemostasis, Oncology, and Stem Cell Transplantation. This study benefitted from access to conserved longitudinal samples collected before Letermovir CMV prophylaxis became standard of care. Two matching control patients showed no CMV reactivation, while six patients had CMV reactivation confirmed quantified via serology and PCR against pp65 test and no other viral infections and reactivation. Both patients without CMV reactivation had no sign of GvHD or other viral infections or reactivations during the observation period (control: ctrl1 and ctrl2). Two patients experienced recurrent reactivation periods (recurrent reactivation: p5 and p6), and four patients had one CMV reactivation prior day 60 after aHSCT (single reactivation: p1, p2, p3, p4). Peripheral blood was obtained at 30, 60, 90 120 and 180 days after aHSCT during regular exams.

All patients and all graft donors had already experienced a primary CMV infection (R +/D +), decreasing the risk for severe CMV reactivation and complication [43, 58, 59] Further patient characteristics are given in Table 1. Patients received MMF until day 50 and cyclosporine A until day 60, which was weaned till day 90. In case of acute GvHD (aGvHD), immunosuppression was prolonged. In three patients, aGvHD periods were documented prior to CMV reactivation, and in one patient after the first CMV reactivation. Two patients were not diagnosed with aGvHD but developed chronic GvHD after the sampling time. Preemptively, all patients were screened for CMV reactivation after aHSCT at least weekly. Upon receiving a positive pp65 result, patients were administered ganciclovir or valganciclovir (dose according to summary of product characteristics). The first CMV reactivation occurred within the first 54 days after aHSCT (34.8 ± 11.7 days) and the first reactivation lasted for 17 days (± 4.62) days and recurrent reactivation lasted between 6 and 24 days (second: 19 ± 5, third 11 ± 5 days) (Table 1, Supplementary Table 1).

### Flow cytometry and cell sorting

Cryopreserved cells were thawed for one minute in a prewarmed 37 °C water bath and gently transferred into 10 ml prewarmed medium (RPMI, 10% FCS,1% penicillin/streptavidin, 1% NaPy, 1%LGlu) and pelleted for 10 min at 1200 rpm. The pellet was resuspended in 1 ml medium and rested for one hour in a 24-well plate at 37 °C in a humid atmosphere containing 5% CO_2_. Following centrifugation, FcBlock (1:10; PBS + 3% FCS) was added for 10 min before cells were stained for 30 min on ice in PBS containing 1% BSA with DAPI (0.1 μg/ml) or LiveDead Discrimination Dye (Zombie NIR, Biolegend) and antibodies against hCD3, hTCRαβ, hTCRγδ and hVδ2 (Supplementary Table 2). In addition, Human TotalSeq-C antibody cocktail was added and stained in parallel (Supplementary Table 2). αβ and γδ T cells were separately sorted, using an Aria Fusion cytometer (BD Biosciences), into low binding tubes containing 500 µl PBS + 3% FCS (Supplementary Fig. 1).

### scRNA-seq and scTCR-seq libraries

Cells from the same donor from available timepoints were hash-tagged and pooled and used for single cell sequencing. Four patients were sequenced in 2020–2022 using 10X Chromium NEXT GEM Single Cell V(D)J Reagent Kits v1.1 using singe indexing method. Here, libraries were prepared for single-cell transcriptome, cell surface protein sequencing and single-cell TCR sequencing were prepared from 20,000 to 30,000 FACS-sorted αβ and γδ T cells using the Chromium Single Cell 5’ Library Gel Bead and Construction kit, along with the Chromium Single Cell V(D)J Enrichment Kit (10 × Genomics, CA, USA), following manufacturer instructions. For the identification of the γδ T cell TCR sequences, following cDNA amplification and quality control, 5 µl were used for γδ TCR sequences using custom primers targeting the TRDC and TRGC gene segments as described previously[30].

First step v1.1:

o Fwd-gd-1: AATGATACGGCGACCACCGAGATCTACACTCTTTCCCTACACGACGCTC
o RV_TRG1: ATCCCAGAATCGTGTTGCTC
o RV_TRD1: CCCACTGGGAGAGATGACAA

Second Step v1.1:

o Fwd-gd-2: AATGATACGGCGACCACCGAGATCT
o RV_TRG2: GGGGAAACATCTGCATCAAG
o RV_TRD2: GACAAAAACGGATGGTTTGG

The transcriptome, cell surface protein and VDJ libraries were sequenced on the Illumina MiSeq or the Illumina NextSeq 500/550 platform at Hannover Medical School.

Samples from further four additional patients were sequences in 2023/24 using Chromium Next GEM Single Cell 5' Reagent Kits v2 (Dual Index). Here, 20 000 to 50 000 FACS-sorted αβ and γδ T cells were used for droplet-based single-cell analysis and transcriptome library preparation using the Chromium Next GEM Single Cell 5' Kit v2, Library Construction kit and Chromium Single Cell Human TCR Amplification Kit according to the manufacturer’s protocols (10 × Genomics). The primers used previously in the v1.1 single-indexing chemistry were adapted according to requirements for the v2 chemistry.

First step v2:

o Fwd-V2-gd: GATCTACACTCTTTCCCTACACGACGC
o RV_TRG1: ATCCCAGAATCGTGTTGCTC
o RV_TRD1: CCCACTGGGAGAGATGACAA

Second Step v2:

o Fwd-V2-gd: GATCTACACTCTTTCCCTACACGACGC
o RV_TRG2: GGGGAAACATCTGCATCAAG
o RV_TRD2: GACAAAAACGGATGGTTTGG

The transcriptome, cell surface protein and VDJ libraries were sequenced on NovaSeq X Plus Series (PE150) or NovaSeq X Plus (PE150-10B) platform.

### Data processing of scRNA-seq libraries

The 10X Cellranger v3.0.2 and Cellranger7.1.0 (10X Genomics) pipeline were used to annotate the Gene-Expression, Cell surface protein and TCR sequences. Following the generation of a cell-gene matric, scRNA-seq reads were aligned to the human reference genome GRCh38 (UCSC, CA, USA).

### Cell clustering

The R package Seurat v5 was used under R v4.4.2 for scRNA-seq data quality control, dimensional reduction, cell clustering, and differential expression analysis. Two separate analyses were done for αβ and γδ T cells. In both analyses first, cells with more than 15% mitochondrial DNA, number of transcripts less than 10 000, and those with less than 200 or over 3,500 features were excluded. Next, Hashtag Oligos and Cell-Surface-Proteins were identified and stored in two separate Object5 Assays. Multiple timepoints per patient were labeled with Total-SeqC Hashtags and sequenced together to avoid batch effects between timepoints. Each sample was individually scaled, clustered, and reduced before integration. After splitting the data by patient, three integration approaches—RPCA, CCA, and Harmony—were evaluated. The method that produced the best biological integration was selected: RPCA for αβ T cells and γδ T cells. Following the integration, layers were joined and the integrated data scaled, dimensional reduction was performed, cluster identified and projected onto a two-dimensional reduction UMAP (Uniform Manifold Approximation and Projection). Following exclusion of CD3low clusters that included cells expressing CD14 and CD20(*MS4A1*), the workflow war repeated. Finally, clustering was performed using serial increase of the resolution 0.1–1 and the final resolution determined by using the package *clustertree* where most clusters appeared to only branch singly and main populations are well defined. The final integration method was determined by the best distribution of patients.

### scTCR-seq analysis

To identify αβ and γδ TCR sequences, cellranger vdj was used using cellranger version 3.0.2 for γδ T cells and version 3.0.2 or 7.1.0 for αβ T cells. Cell Ranger vdj (10 × Genomics) was performed to generate scTCR annotations. Human genome GRCh38 (refdata-cellranger-vdj-GRCh38-alts-ensembl-3.1.0 and refdata-cellranger-vdj-GRCh38-alts-ensembl-7.0.0) were used as the reference for alignment. Nonproductive TCR sequences and pseudogenes were excluded, and single and paired TCRs were identified based on identical barcodes. As the libraries for scTCR and scRNA-Seq were prepared separately but same cells share the same barcode, we included only those TCR sequences of cells that passed the quality control of the scRNA-seq analysis. γδ T cells of p6 were excluded from further analysis due to a failed chip load (Supplementary table ST3). Paired or single TCR chain sequences were defined as “public” if identified in more than one patient. Clonal frequencies were calculated for each clone, timepoint and patient individually. Repertoire diversity was measured using the Gini index (ineq), and repertoire similarity between samples and timepoints was quantified using the Morisita-Horn index (vegan), with both metrics incorporating clone abundance distributions.

### Statistics

Longitudinal changes across post-transplant timepoints (day 30, 60, 90, 120, and 180) were analyzed in R using linear mixed-effects models to account for repeated measurements within individuals and missing timepoints. For each outcome (e.g., frequencies of non-Vδ2 γδ T cells and repertoire-defined non-TRDV2, values were summarized per individual and timepoint as percentages of all cells with non-missing annotation for the respective label. No imputation was performed; observations with missing values for the variable of interest were excluded from the corresponding analysis only. For each outcome, a linear mixed-effects model of the form value∼day + (1∣patient) was fitted using the lme4 package, with timepoint modeled as a fixed effect and patient ID as a random intercept. Significance of the overall time effect was assessed using F-tests with Kenward–Roger degrees-of-freedom approximation as implemented in lmerTest. When the overall time effect was detected, pairwise post-hoc comparisons between timepoints were performed on estimated marginal means (emmeans) with Bonferroni correction for multiple testing. Unless stated otherwise, adjusted two-sided p-values < 0.05 were considered statistically significant.

For each clone of interest, a 2 × 2 contingency table was constructed at each timepoint comparing: (i) clone of interest versus all other clones, and (ii) cells within the target cluster versus other clusters. Target clusters of interest (c01 and c07 were combined into c01_7 and clusters c04, c06 and c08 into c04_6_8) were defined based on phenotypic characteristics. Fisher's exact test was employed to assess clone enrichment within the target cluster. Odds ratios and log2-transformed odds ratios were calculated as measures of effect size. P-values were adjusted for multiple comparisons across clones and timepoints using the Bonferroni correction. Significance thresholds were defined as: *p < 0.05.

### Visualization

Visualizations were generated in R using ggplot2. Longitudinal data were displayed as individual trajectory plots, with lines connecting available timepoints per patient and consistent subject-specific color coding across figures. Proportions of cells within target clusters were plotted for clones of interest and other clones across timepoints, with statistical significance annotated directly on plots. Compositional changes of categorical subsets were visualized using stacked bar charts and alluvial plots, based on aggregated counts or relative frequencies per patient and timepoint. A standardized compact theme was applied to ensure consistent formatting across all figures.

## Supplementary Information

Below is the link to the electronic supplementary material.

Supplementary file1 (XLSX 13 KB)

Supplementary file2 (XLSX 11 KB)

Supplementary file3 (XLSX 12 KB)

Supplementary file4 (DOCX 2808 KB)

## Data Availability

The scRNAseq and scTCRseq data are available at Zenodo (10.5281/zenodo.21409822).
