# Supplementary material for: γδ T cells show distinct responses to CMV after stem cell transplantation
Source: J Mol Med (Berl). 2026 Jul 20;104(1):96. doi: 10.1007/s00109-026-02703-8 (PMC13381381; doi:10.1007/s00109-026-02703-8)
Supplement: Supplementary file 4 — Supplementary file4 (DOCX 2808 KB) [file 109_2026_2703_MOESM4_ESM.docx]

**Supplementary Materials** for manuscript JMME-D-26-00273:

TCR barcodes track distinct anti-CMV responses
of γδ T cell clones after stem cell transplantation

Authors: Freya Sibbertsen, Zheng Song, Cedric Ly, Inga Sandrock, Stefan Bonn, Christian Koenecke, Likai Tan, Christian Schultze-Florey, Immo Prinz^*^

**Contents**

[Supplementary Figure 1: Flow cytometry gating strategy for the sort of αβ and γδ T cells. 2](#_Toc224209465)

[Supplementary Figure 2: Private delta and beta but public gamma and alpha chains. Rare public TRG expression, rare overlap in TRA, TRB, and TRD. 3](#_Toc224209466)

[Supplementary Figure 3: Frequencies of γδ T cell subsets over time. Flowcytometry and scRNA analyses. 4](#_Toc224209467)

[Supplementary Figure 4: Monitoring γδ TCR repertoire stability within patients over 180 days. 5](#_Toc224209468)

[Supplementary Figure 5: Monitoring γδ T cell phenotypes and TCR delta chain usage within patients over 180 days. 6](#_Toc224209469)

[Supplementary Figure 7: Monitoring γδ T cell phenotypes of Vγ9Vδ2^+^ T cells within patients over 180 days. 8](#_Toc224209470)

[Supplementary Figure 8: Clonal expansion and phenotypic shift of individual clones. 9](#_Toc224209471)

[Supplementary Figure 9: Monitoring αβ T cell phenotypes within patients over 180 days. 10](#_Toc224209472)

[Supplementary Figure 10: Monitoring αβ TCR repertoire stability within patients over 180 days. 11](#_Toc224209473)


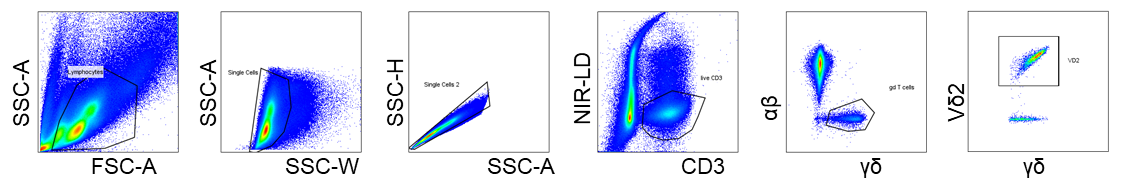


## **Supplementary Figure 1: Flow cytometry gating strategy for the sort of αβ and γδ T cells.**

## **
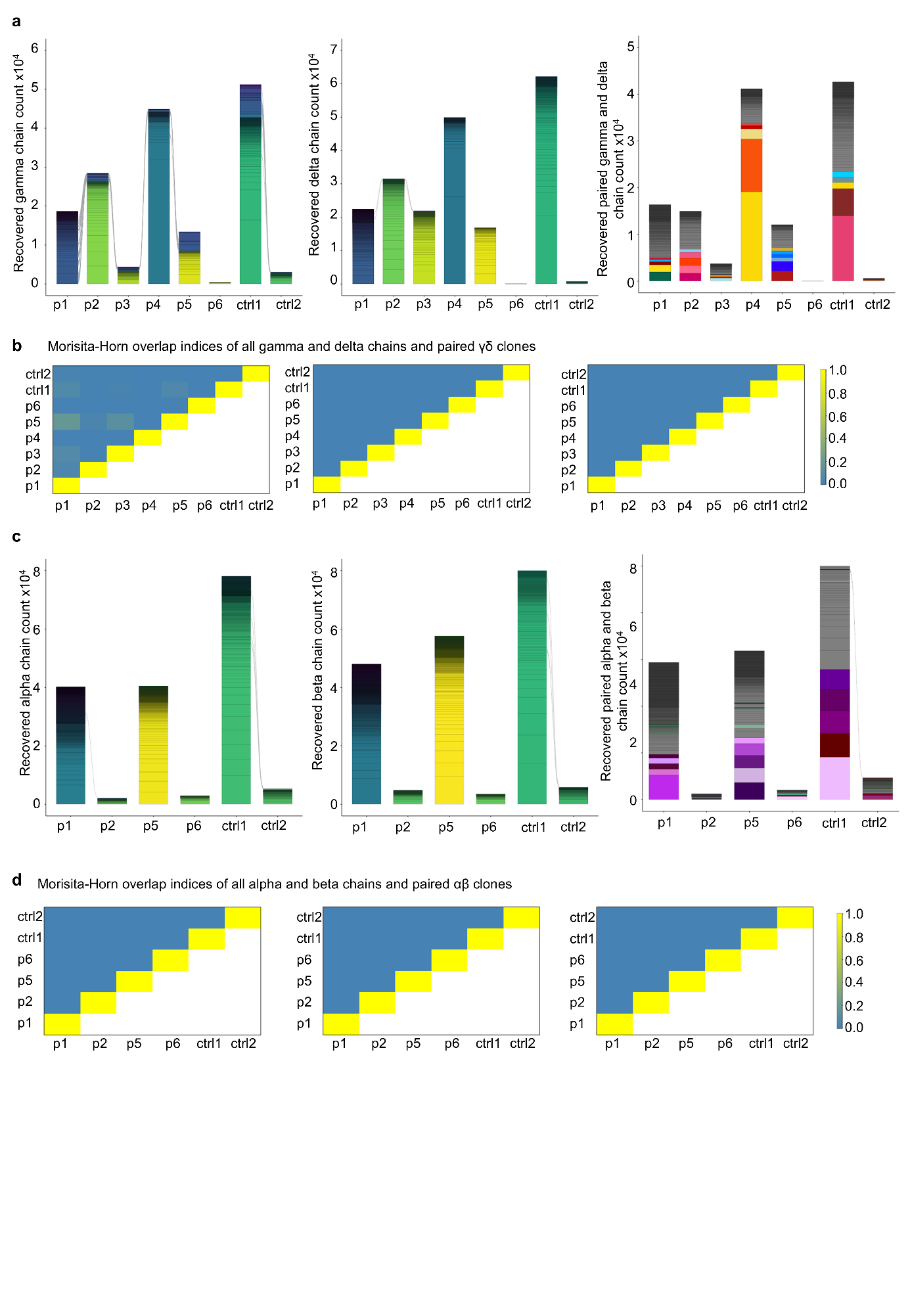
**

## **Supplementary Figure 2: Private delta and beta but public gamma and alpha chains. Rare public TRG expression, rare overlap in TRA, TRB, and TRD.**

**a**) Overlap of paired, gamma and delta, chains in patients with (p1-p6, n= 6) and without (ctrl1, ctrl2, n=2) CMV reactivation. **b**) Interpatient Morisita-Horn indices for all gamma and delta chains and paired clones. 1 indicates identical samples and 0 no clonal overlap. **c**) Overlap of alpha and beta, chains and paired clones in patients with (p1, 3, 5, 6 n= 4) and without (ctrl1, ctrl2, n=2) CMV reactivation. d) Interpatient Morisita-Horn Index for all alpha and beta chains and paired clones. 1 indicates identical samples and 0 no clonal overlap.

##
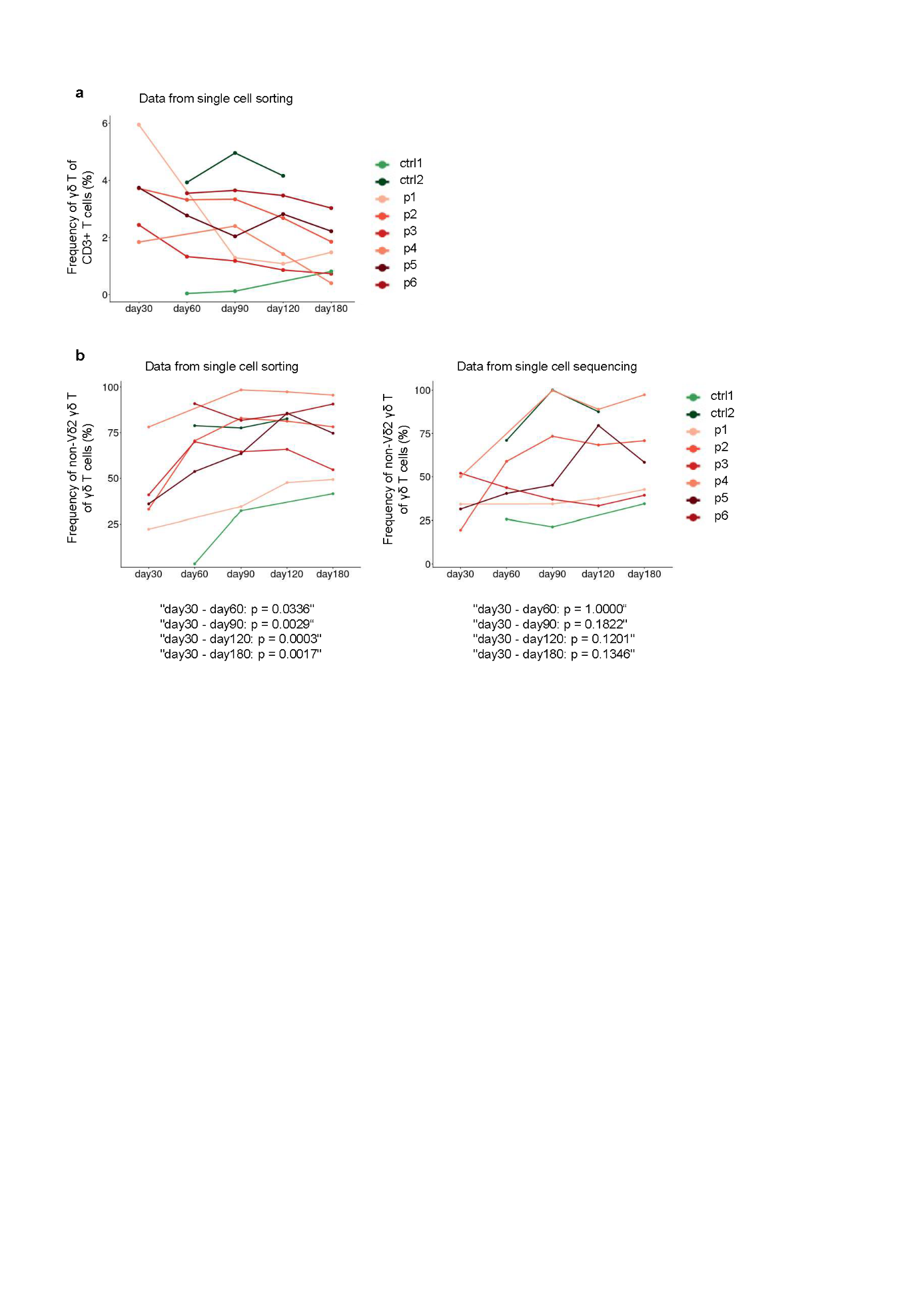


## **
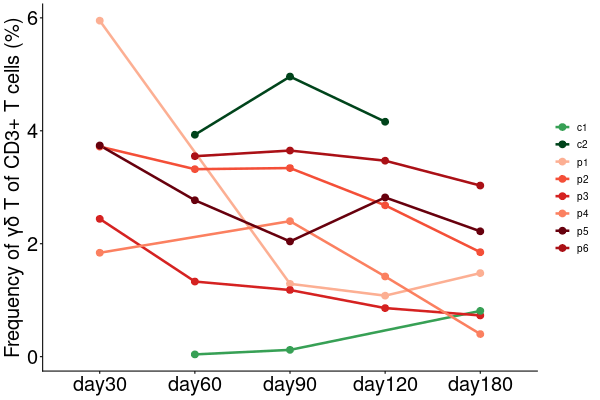

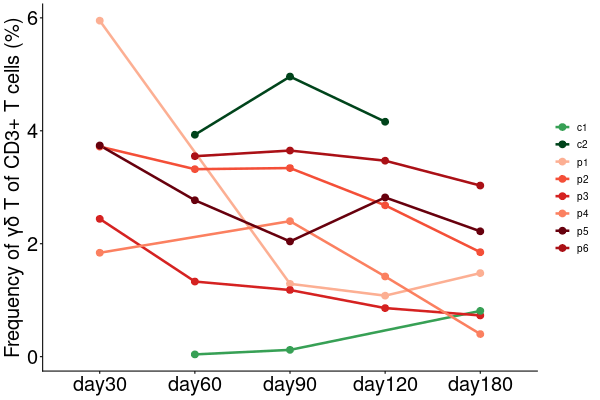
Supplementary Figure 3: Frequencies of γδ T cell subsets over time. Flowcytometry and scRNA analyses.**

Frequency of γδ and non-Vδ2^+^ γδ T cells across various patient samples. **a**) Percentage of γδ cells of all CD3^+^ T cells. **b**) Frequency of Vδ2^+^ γδ T cells from sorting analysis of γδ T cells per day, focusing solely on delta chains. The right panel represents the frequency of Vδ2^+^ T cells from derived from single-cell sequencing from all recovered delta chains at the indicated timepoint. Each line represents a different patient, with markers indicating the respective patient samples (p1-p6 and ctrl1 and ctrl2). Statistical comparisons (day 30 vs. subsequent timepoints) were performed for CMV-reactivated patients using linear mixed-effects modeling with Bonferroni correction. p-values shown below the graph correspond to pairwise comparisons between day 30 and subsequent timepoints, derived from linear mixed-effects modeling with Bonferroni correction for multiple testing.

## **
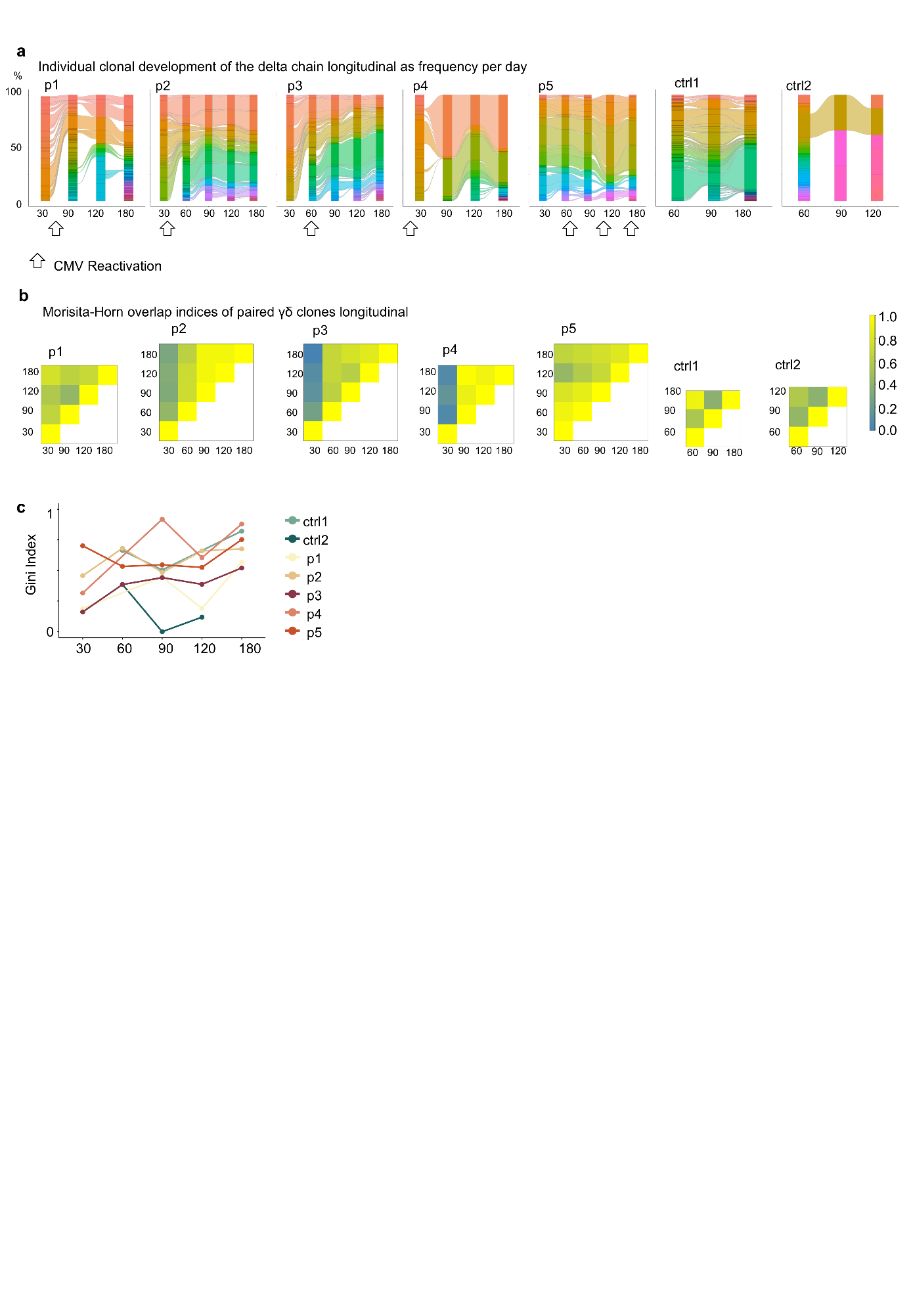
Supplementary Figure 4: Monitoring γδ TCR repertoire stability within patients over 180 days.**

**a**) Longitudinal frequency of delta chains. The x-axis represents the time points of available samples, while the y-axis shows the percentage of all delta chains available per day. The clonal development of delta chains (paired and unpaired with a gamma chain) is presented for each patient individually. **b**) Intra-patient Morisita-Horn indices for paired γδ clones longitudinally. 1 indicates identical samples and 0 no clonal overlap. **c**) Gini Indices per patient longitudinal. Values range from 0 (perfect equality, all clones at equal frequency) to 1 (perfect inequality, single clone dominance). The black and white arrow indicates CMV reactivation


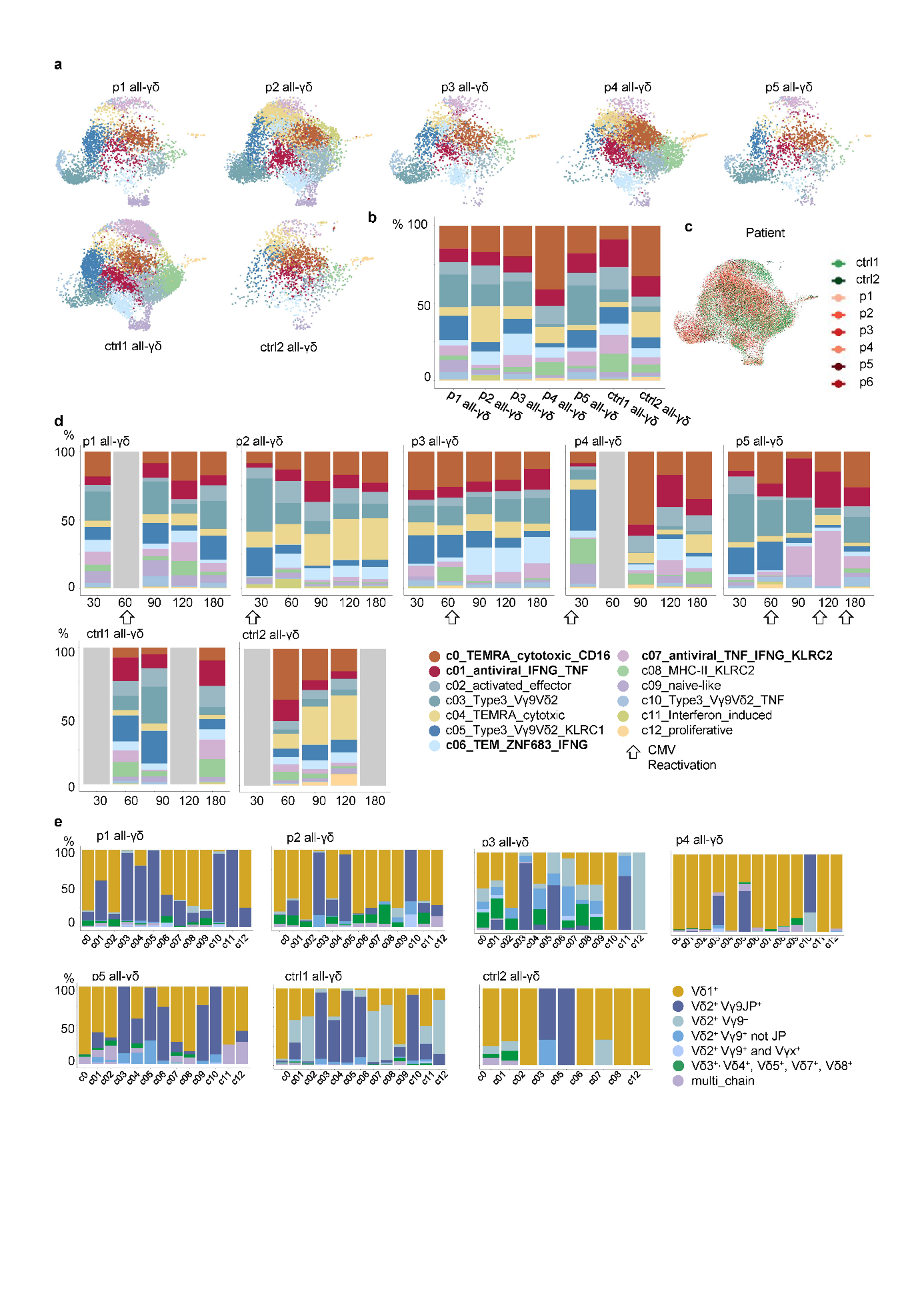


## **Supplementary Figure 5: Monitoring γδ T cell phenotypes and TCR delta chain usage within patients over 180 days.**

**a**) UMAP visualization of the integrated γδ T cell dataset shown separately for each patient. **b**) Relative cluster composition (c0–c12) per patient displayed as proportional bar plots. **c**) UMAP colored by patient identity. **d**) Longitudinal cluster distribution across time points for each patient; arrows indicate CMV reactivation events. Each color represents a distinct cluster identified by unsupervised clustering (Figure 2a). **e**) Bar plots show the distribution of major γδ T cell subsets (based on Vδ chain) across clusters for each patient and control. The black and white arrow indicates CMV reactivation. Grey bars indicate no sample available.


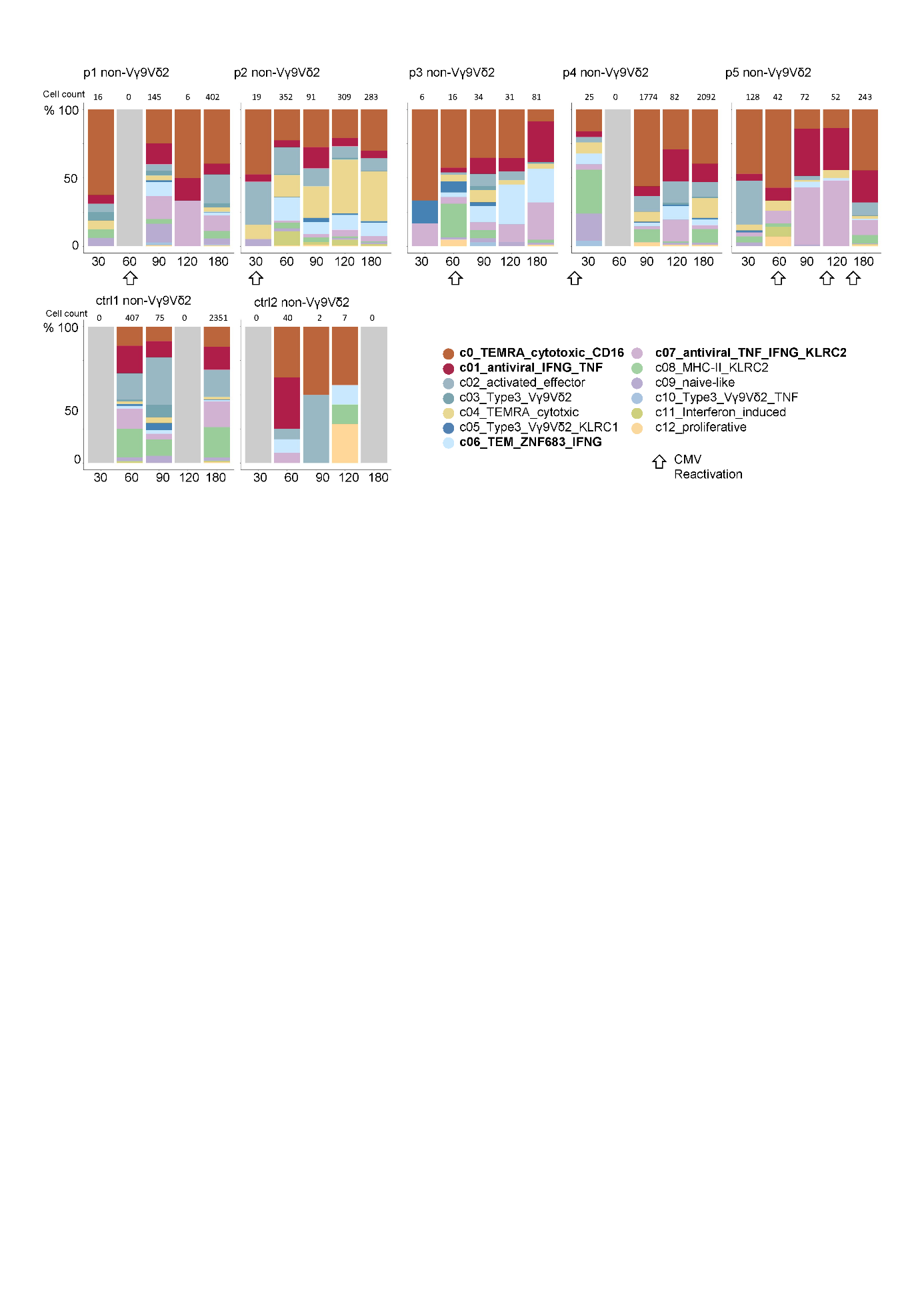


**Supplementary Figure 6: Monitoring γδ T cell phenotypes of adaptive-like γδ T cells within patients over 180 days.**

Cell distribution of all non-Vγ9Vδ2 γδ T cell clusters longitudinally for each patient individually at available time points following transplantation. Stacked bar plots show the proportion of γδ T cells per identified cluster over time (days post-transplant or post-treatment, as indicated). Each color represents a distinct cluster identified by unsupervised clustering. Each color within the bars corresponds to a specific T cell cluster described in Figure 2a. The black and white arrow indicates CMV reactivation. Grey bars indicate no sample available.

## **
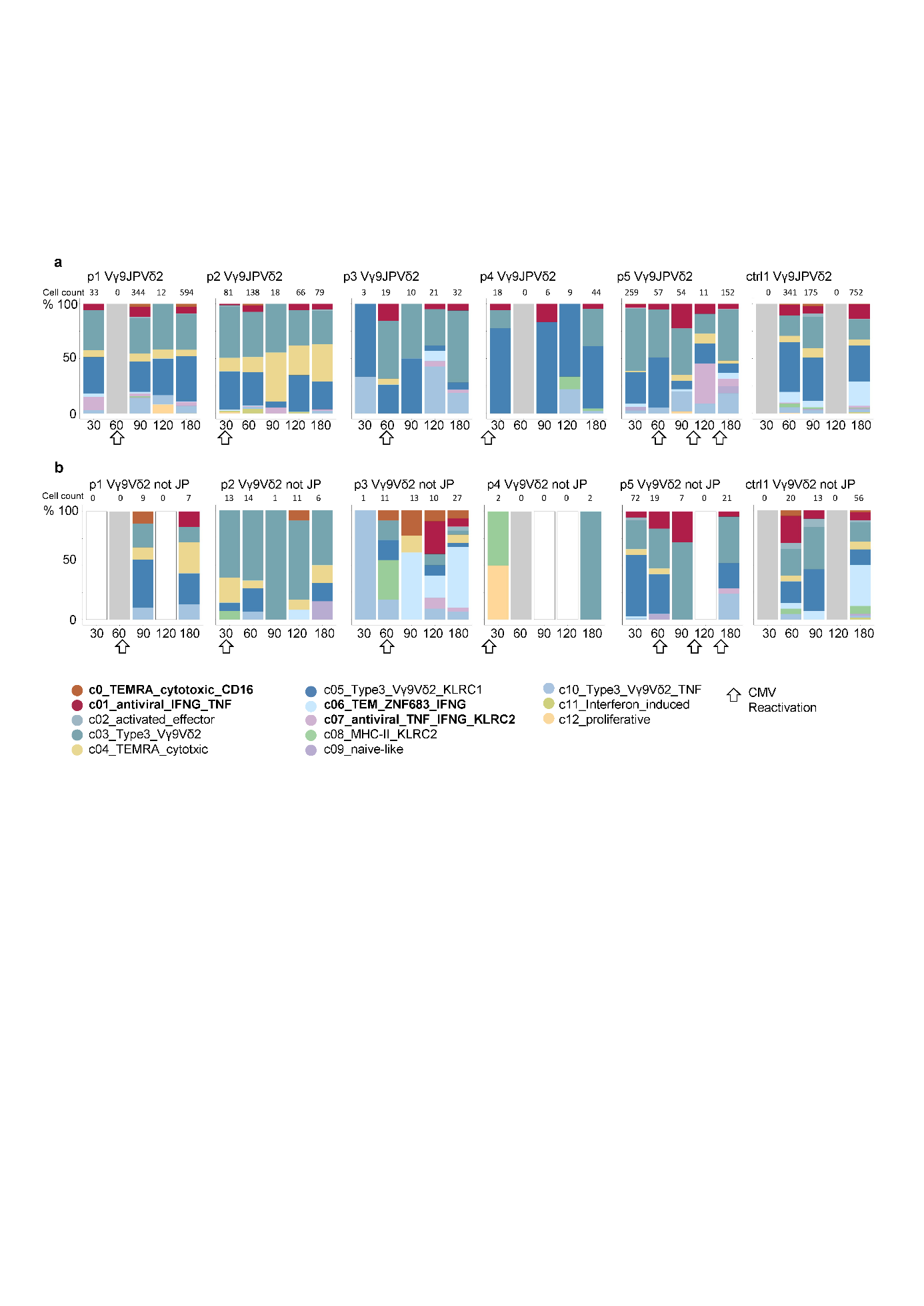
Supplementary Figure 7: Monitoring γδ T cell phenotypes of Vγ9Vδ2^+^ T cells within patients over 180 days.**

Cell distribution of a) Vγ9Vδ2^+^ T cells using JP VJ connection and b), Vγ9Vδ2^+^ T cells using no JP VJ connection longitudinally for each patient individually at available time points following transplantation. Stacked bar plots show the proportion of γδ T cells per identified cluster over time (days post-transplant or post-treatment, as indicated). Each color represents a distinct cluster identified by unsupervised clustering (Figure 2A). Each color within the bars corresponds to a specific T cell cluster described in Figure 2a. In ctrl2, too few Vγ9Vδ2^+^ T cells were recovered to be analyzed. The black and white arrow indicates CMV reactivation. Grey bars indicate no sample available, a white bar with black border indicates available sample but no cell of interest detected.

##
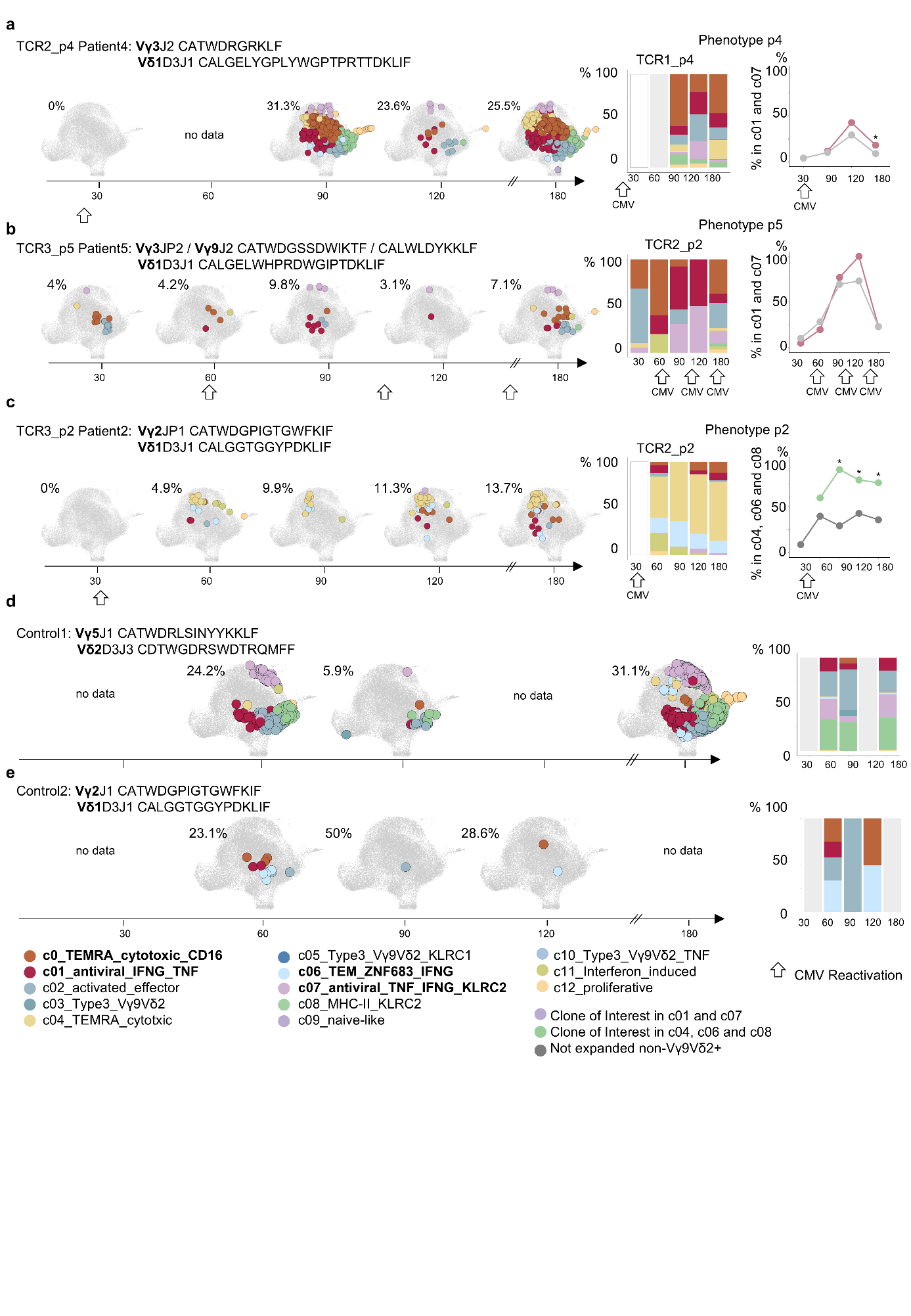
**Supplementary Figure 8: Clonal expansion and phenotypic shift of individual clones.**

Phenotype of three individual clones of interest from **a**) patient4, **b**) patient5 and **c**) patient2, **d**) control1, **e**) control2. Tracking the phenotype of the clone of interest longitudinally in the UMAP. Highlighted dots represent one cell of the indicated clone. All clones share the same paired CDR3 sequence. The frequency of the clone of interest per patient per day is indicated above each UMAP. Bar chart representation of the phenotype of the clone of interest longitudinally (left side). Line plots show the proportion of cells within the cluster (c01 and c07 – red or c04, c06, c08 - green) for clones of interest versus all other clones (grey) across post-transplant timepoints. Significance stars indicate Bonferroni-adjusted Fisher's exact test p-values (*p<0.05) (right side). Cells are coloured based on the associated UMAP clusters (Figure 2a). The black and white arrow indicates CMV reactivation. Grey bars indicate no sample available.

##
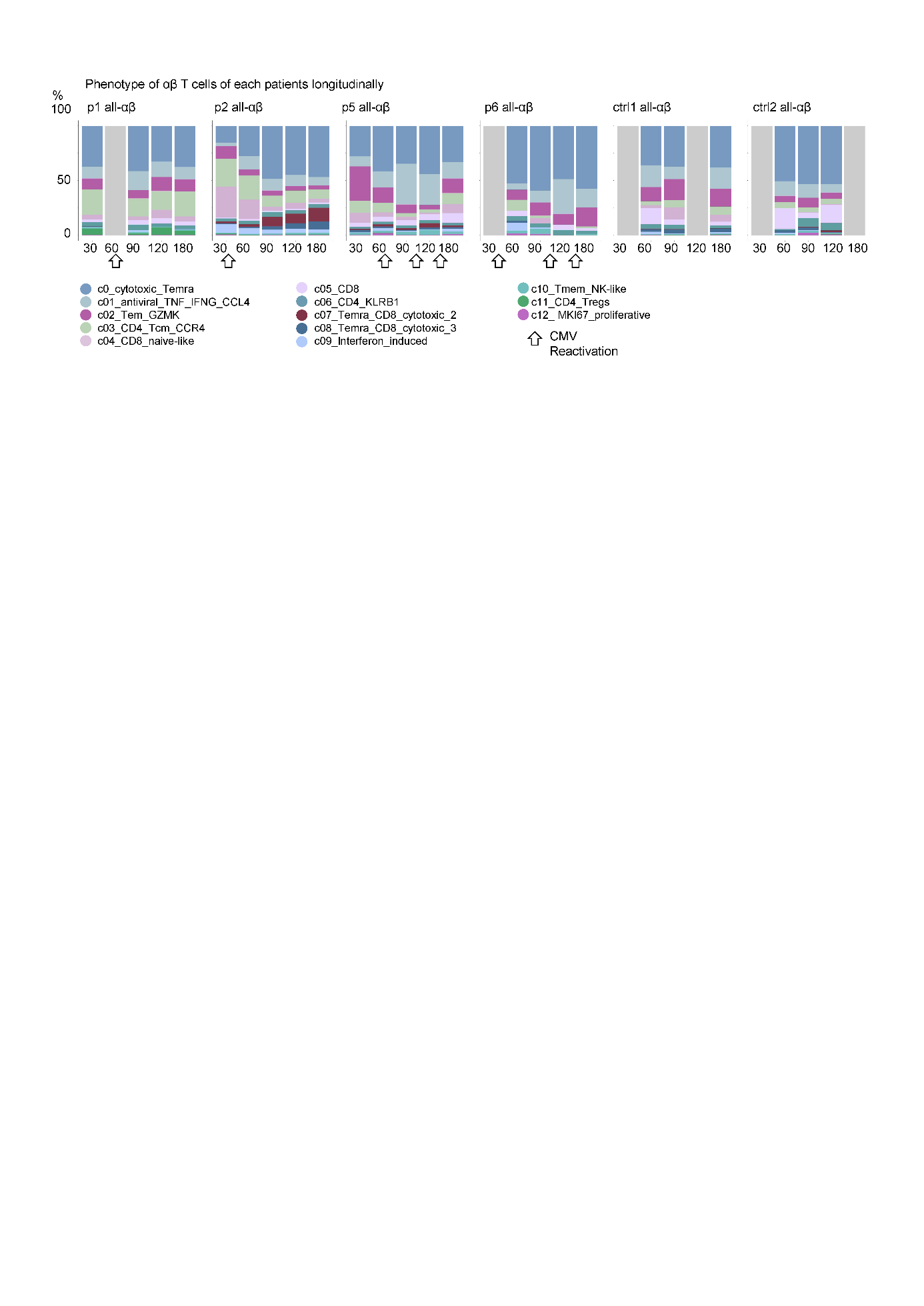
**Supplementary Figure 9: Monitoring αβ T cell phenotypes within patients over 180 days.**

Cell distribution of all αβ T cell clusters longitudinally for each patient individually at available time points following transplantation. Stacked bar plots show the proportion of αβ T cells per identified cluster over time (days post-transplant or post-treatment, as indicated). Each color represents a distinct cluster identified by unsupervised clustering (Figure 6a). Each color within the bars corresponds to a specific T cell cluster described in Figure 6a. The black and white arrow indicates CMV reactivation. Grey bars indicate no sample available.


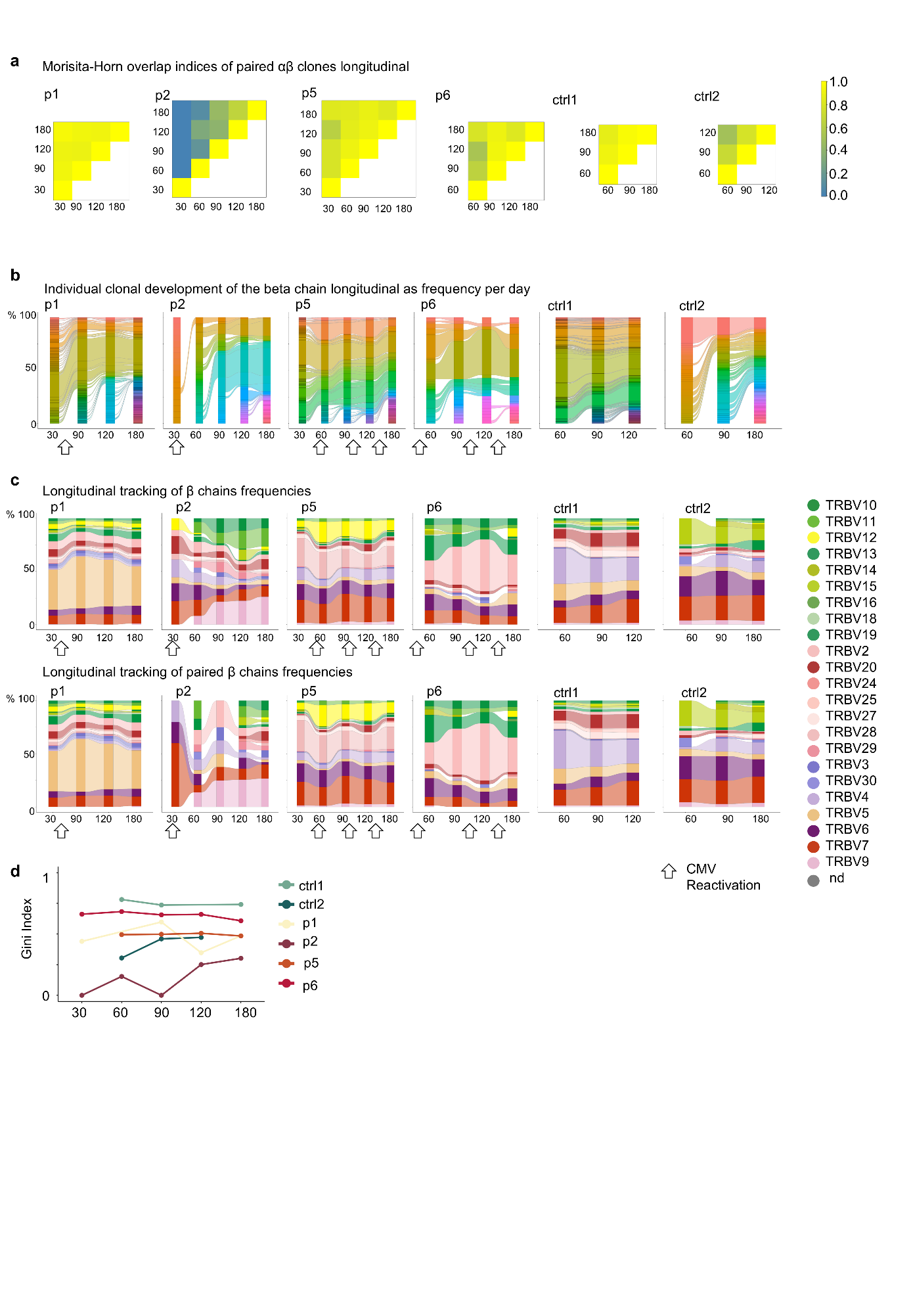


## **Supplementary Figure 10: Monitoring αβ TCR repertoire stability within patients over 180 days.**

**a**) Intra-patient Morisita-Horn indices for paired αβ clones longitudinally. 1 indicates identical samples and 0 no clonal overlap. **b**) Longitudinal frequency of beta clones. The x-axis represents the time points of available samples, while the y-axis shows the percentage of all beta chains available per day. The clonal development of beta chains (paired and unpaired with an alpha chain) is presented for each patient individually. **c**) Frequency of individual TRBV beta chains per patient longitudinally. The x-axis represents the time points of available samples, while the y-axis shows the percentage of all beta chains available per day. Overlap of beta chains (paired and unpaired with an alpha chain) is presented for each patient individually. The black and white arrow indicates CMV reactivation. **d**) Gini Indices per patient longitudinal. Values range from 0 (perfect equality, all clones at equal frequency) to 1 (perfect inequality, single clone dominance).
